# Comparison of different macroinvertebrates bioassessment indices in a large near‐natural watershed under the context of metacommunity theory

**DOI:** 10.1002/ece3.10896

**Published:** 2024-02-05

**Authors:** Guohao Liu, Xinxin Qi, Zongwei Lin, Yuanyuan Lv, Sangar Khan, Xiaodong Qu, Binsong Jin, Ming Wu, Collins Oduro, Naicheng Wu

**Affiliations:** ^1^ Department of Geography and Spatial Information Techniques Ningbo University Ningbo China; ^2^ Zhejiang Collaborative Innovation Center & Ningbo Universities Collaborative Innovation Center for Land and Marine Spatial Utilization and Governance Research Ningbo University Ningbo China; ^3^ State Key Laboratory of Simulation and Regulation of Water Cycle in River Basin China Institute of Water Resources and Hydropower Research Beijing China; ^4^ College of Life and Environmental Sciences Hangzhou Normal University Hangzhou China; ^5^ Wetland Ecosystem Research Station of Hangzhou Bay, Research Institute of Subtropical Forestry Chinese Academy of Forestry Hangzhou China

**Keywords:** aquatic insects, biomonitoring, biotic index, dispersal processes, freshwater ecosystems, stream, trait

## Abstract

The metacommunity theory proposes that community structure and biodiversity are influenced by both local processes (such as environmental filtering) and regional processes (such as dispersal). Despite the extensive use of traditional bioassessments based on species‐environment relationships, the impact of dispersal processes on these assessments has been largely overlooked. This study aims to compare correlations between various bioassessment indices, including Shannon Weiner (H′), Biological Monitoring Working Party (BMWP), average score per taxon (ASPT), biotic index (BI), and EPT taxa index (EPT), based on macroinvertebrates collected from 147 sampling sites in a subtropical Chinese near‐natural catchment. Modified indices were calculated by removing species strongly influenced by dispersal processes to address the influence of dispersal processes. Their relationship with environmental factors was then compared to the original indices. The study employed random forest regression (RFR) to compare the explanatory power of environmental factors using the two sets of indices. The spearman rank correlation analysis was conducted to examine the correlation between indices and environmental factors. The river health assessment was performed based on both modified and original indices. The results reveal significant differences between original and modified indices (especially H′ and BI) providing a more accurate reflection of environmental conditions. Furthermore, the sensitivity of the different indices to various environmental factors varied, leading to differences in the bioassessment results between the modified and the original indices. Notably, original H′, BMWP, and ASPT overestimated the bioassessment results, whereas the original BI underestimated them. These findings offer valuable insights into bioassessment and river health assessment evaluation within the catchment and other interconnected freshwater ecosystems, such as lakes, reservoirs, and wetlands. Our study underscores the importance of assessing and mitigating the impact of dispersal processes on bioassessment to obtain a more precise representation of the status of freshwater ecosystems.

## Graphical abstract

Assessment of the impact of dispersal processes on macroinvertebrate bioassessment indices. Comparison of the response of different bioassessment indices to environmental factors.

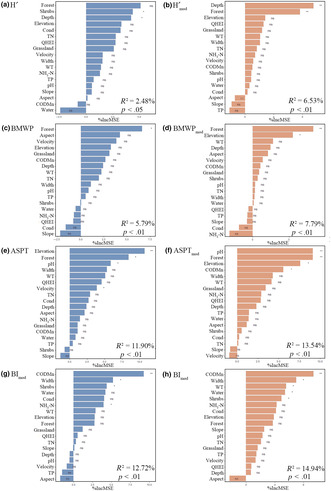

## 1 INTRODUCTION

The ecological importance of freshwater ecosystems, such as rivers and streams, cannot be ignored as they are irreplaceable resources that play a critical role in human and social development (Carpenter et al., 2011; Luo et al., 2018). However, freshwater ecosystems face threats from stressors like overexploitation, habitat fragmentation, agricultural activities, and land use changes (Grill et al., 2019; Kundzewicz et al., 2008; Moss, 2008). The impact of these stressors has led to a decline in biodiversity and loss of ecosystem function in freshwater ecosystems (Albert et al., 2021). In order to protect, manage, and restore freshwater biodiversity and associated natural resources the development and validation of diverse bioassessment methodologies are very important (Angeler et al., 2014; Wu et al., 2020). The key idea of bioassessment is that changes in the biological communities found in different locations are a reflection of the environmental conditions between sites (Vilmi et al., 2016). But metacommunity theory suggests that the composition of biological communities is a combination of local (e.g., environmental filtering), regional (e.g., dispersal), and stochastic (e.g., random local disturbances) processes (Cottenie, 2005; Leibold et al., 2004). Therefore, there is a need to validate and compare traditional bioassessments based on species–environmental relationships to provide more accurate information on the ecological health of freshwater ecosystems.

Using aquatic organisms for freshwater ecosystem health assessment is a well‐established approach as they are affected by a wide range of stressors that reflect the effects of pollution conditions and changes in habitat and hydrological conditions (Hering et al., 2006; Wu et al., 2023). To date, bioassessment with various aquatic organisms (e.g., fish, macroinvertebrates, and algae) is widely used in many countries and organizations, such as the European Water Framework Directive (WFD) and the US National Biomonitoring Program (NBP) (Arle et al., 2016; Buss et al., 2015). Bioassessment indices are the key tool for bioassessment of freshwater biomonitoring. Their usage proposed information on environmental health, which plays an important role in defining the objectives of environmental management and related policies (Wu et al., 2012; Zhao et al., 2019). Macroinvertebrates are widely used in bioassessment due to their suitable life history, habitat fixation, ease of collection, and ability to rapidly respond to various stressors such as water pollution and anthropogenic influences (Feld & Hering, 2007; Huang et al., 2015; Mereta et al., 2013). The bioassessment indices for macroinvertebrates mainly include: (1) biodiversity indices such as the Shannon Weiner (H′) and Simpson index (Linares et al., 2020); (2) taxonomic unitarity indices such as the EPT taxa index (EPT) (Kitchin, 2005); and (3) indices based on tolerance or sensitivity values for macroinvertebrates such as the BI (biotic index), FBI (family‐level biotic index) (Young et al., 2014), BMWP (Biological Monitoring Working Party), and improved ASPT (average score per taxon) index based on BMWP (Armitage et al., 1983; Ruse, 1996). However, these traditional bioassessment indices all share a common problem: they ignore the impact of spatial processes on communities (Vilmi et al., 2016).

Metacommunity theory suggests that environmental factors are not the only factors that define the changes in community structure, but spatial processes are also one of the important processes that affect community structure (Heino et al., 2015; Lin et al., 2024; Thompson et al., 2020; Wu et al., 2022). Many studies suggested the impact of dispersal processes (i.e., mass effect and dispersal limitation) on biodiversity (De Bie et al., 2012; Li et al., 2021; Tonkin et al., 2018). Mass effect assumes that environmentally heterogeneous habitat patches are tightly linked through dispersal such that colonization in source habitats can persist in sink habitats (Mouquet et al., 2003; Mouquet & Loreau, 2002). Dispersal limitations prevent species from reaching suitable habitats (Heino et al., 2015). However, many commonly used indices are often based on traditional assessment concepts (i.e., species‐environment relationships) that ignore many important ecological processes (Leboucher et al., 2021; Siqueira et al., 2014). Spatial processes may isolate the relationship between species and their environment (Heino et al., 2017), thus affecting the results of biological assessments. Therefore, the ability of bioassessments to accurately reflect local environmental conditions may be challenged in metacommunity contexts (Bried & Vilmi, 2022; Leboucher et al., 2021). Hence, improvements and comparisons of different bioassessment indices are imminent.

**FIGURE 1:**
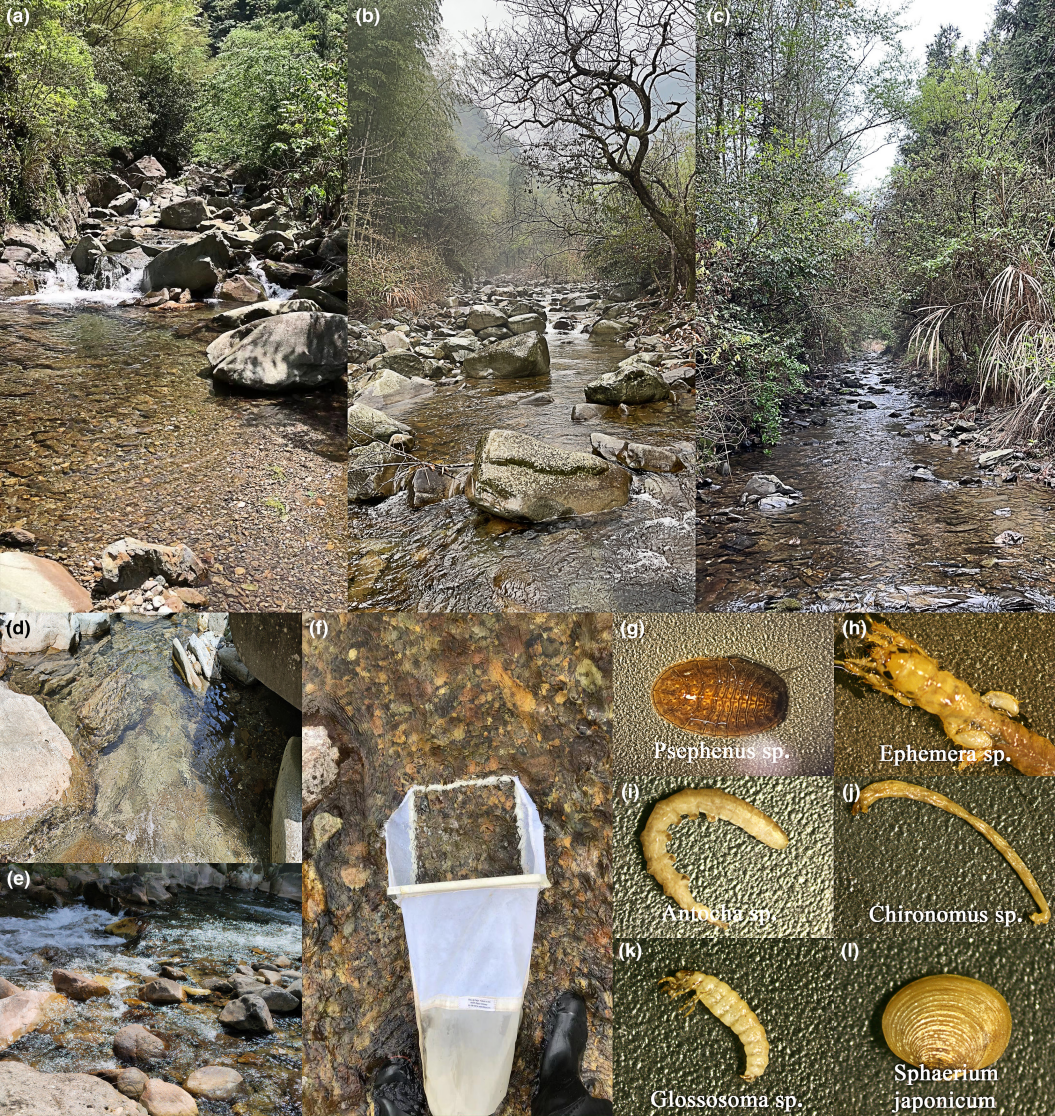
Habitat conditions and examples of macroinvertebrates in the Thousand Island Lake (TIL) catchment. (a–c) Different habitats, (d, e) microhabitats with different water flow conditions (slower and faster flows), (f) Surber net and (g–l) examples of typical macroinvertebrates.

The samples were taken from Thousand Islands Lake (TIL) catchment in China. This area spans the administrative regions of Hangzhou and Huangshan and primarily consists of the Xin'an River and its tributaries (Gu et al., 2016). TIL holds a critical ecological position due to being the largest artificial freshwater reservoir in China and an important source of drinking water for the Yangtze River Delta region (Sheng & Han, 2022b). Characterized by mountainous terrain with extensive forest cover, this region experiences limited anthropogenic activity, predominantly agriculture. Effective control of industrial and domestic wastewater has led to well‐preserved ecosystems and biomes (Figure 1, Lin et al., 2024; Wang et al., 2012; Zhai et al., 2014). These factors make it an ideal area for studying the impact of quantitative dispersal processes on bioassessment indices in a near‐natural context. We selected several different extant bioassessment indices of H′, BMWP, ASPT, BI, and EPT. Different traits of macroinvertebrates are affected by dispersal processes to different degrees (Lindholm et al., 2018), especially traits related to dispersal ability, thus the dispersal process may separate the link between organisms and the environment and thus affect bioassessment. The species that are more influenced by spatial processes were removed according to a previous study (Liu et al., 2023), to calculate the corresponding modified indices (hereafter as index_mod_, respectively). Here we compare the index_mod_ with the original index in an attempt to address the impact of the dispersal process on bioassessment.

With this study, our goals were: (1) to evaluate the differences in correlations between the original indices and indices_mod_ with environmental factors; (2) to compare the propensity of different bioassessment indices to indicate the role of various environmental factors to provide useful information for bioassessment and river health management; and (3) to test differences in the water health assessment classes between the two version of the indices.

## 2 MATERIALS AND METHODS

### 2.1 Study area description

**FIGURE 2:**
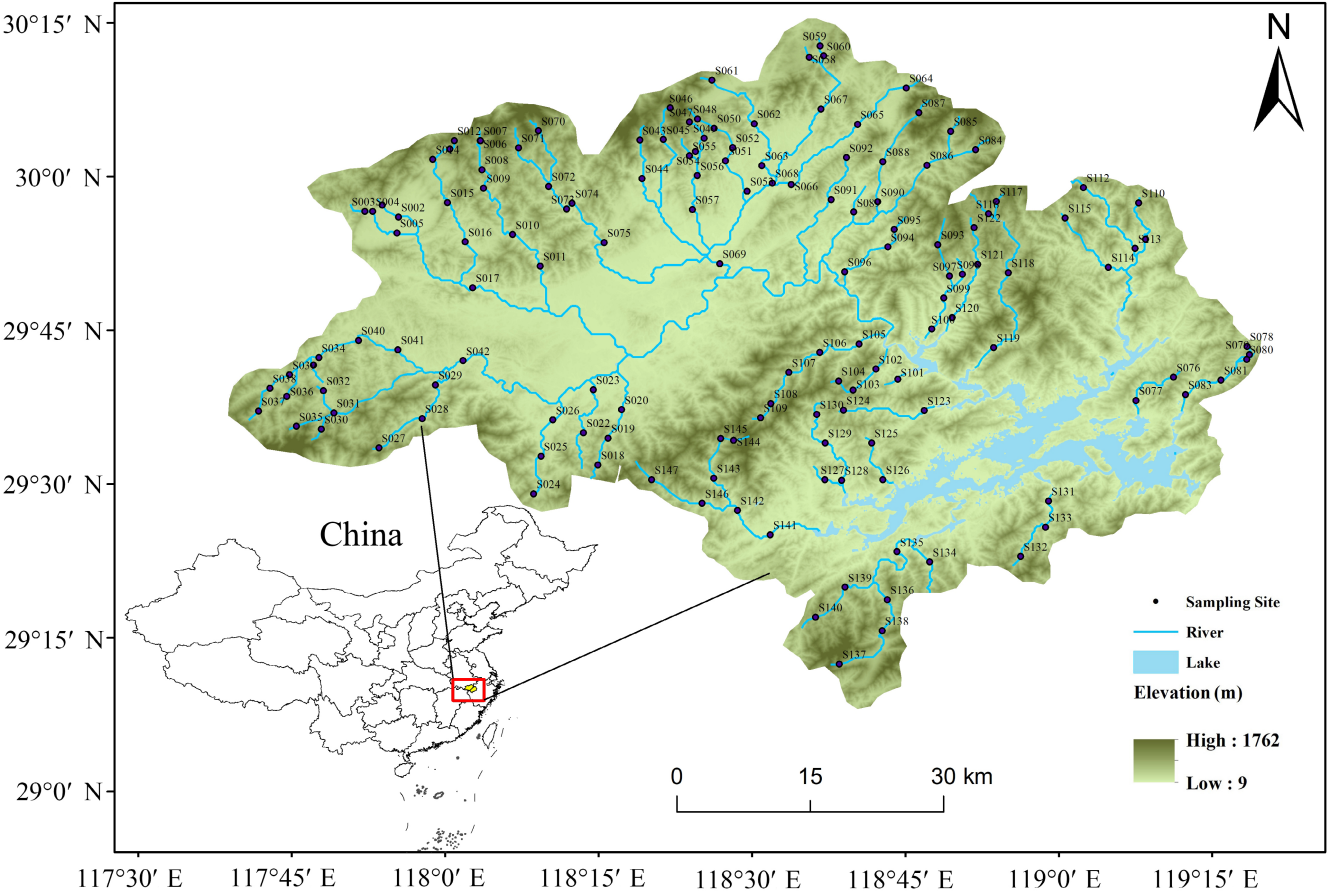
Distribution map of the 147 sampling sites in the Thousand Islands Lake (TIL) catchment, China.

The study encompassed 147 sampling sites within the Thousand Islands Lake (TIL) catchment area (29.21°–30.21° N; 117.70°–119.31° E; elevation gradient over 500 m), China (Figure 2). Situated within a subtropical monsoon climate zone, this sampling region spans an approximate area of 10,080 km^2^ and experiences an average annual precipitation of 1430 mm. Sampling sites are designed to avoid locations with rapidly fluctuating water quality (e.g., wastewater outfalls) and important hydraulic structures (e.g., bridges, water intakes). At the same time, the habitats in the area are well protected, with low anthropogenic activity and low gradients of environmental disturbance.

### 2.2 Macroinvertebrates data

The field investigation was conducted in the dry season (April and May 2021) to avoid heavy rainfall in the TIL catchment. We used Surber net (Figure 1f, 30 × 30 cm^2^, 500 μm mesh) for three replicate collections at each sampling site. Different microhabitats (Figure 1a–c, mainly fine sediments, rocks, and pebbles) and flow conditions (Figure 1d,e, slower and faster flows) were sampled to cover as many species as possible. Finally, three samples were combined to represent the macroinvertebrates at the site. The sampling points cover the wadable section of the river from the headwaters to the main river. The samples were stored in a removable holding tank and preserved in 70% alcohol for further identification. The macroinvertebrates were classified at the lowest possible taxonomic level (Figure 1g‐l, mainly genera) using references (Holzenthal, 2009; Merritt et al., 2017; Morse et al., 1994) and online identification resources (https://www.macroinvertebrates.org/).

### 2.3 Environmental factors

A comprehensive set of 12 physicochemical parameters was collected at each of the sampling sites, as outlined in Table 1. The assessment of hydro‐morphological characteristics encompassed the measurement of river width (Width), water depth (Depth), and flow velocity (Velocity). For these measurements, we used the Global Water Flow Probe FP201 to measure Depth and Velocity in the vicinity of the sampling locations and to measure Width at the river transects where they were located. Each site was assessed and scored according to Qualitative Habitat Evaluation Index (QHEI) (Taft & Koncelik, 2006). The QHEI was calculated for six metrics: substrate, instream cover, channel morphology, riparian zone and bank erosion, pool/glide and riffle‐run quality, and map gradient. Each category was assessed visually and assigned a score from 0 to 20, with the final QHEI score being the sum of these six categories. The pH, conductivity (Cond), and water temperature (WT) were measured by using a water quality handheld meter (YSI Professional Plus). In addition, water samples were collected at each site by using 100 mL sample bottle and stored at 4°C in the laboratory for further analysis. The quantification of total phosphorus (TP), total nitrogen (TN), ammonia nitrogen (NH_3_–N), nitrate nitrogen (NO_3_–N), and chemical oxygen demand (CODMn) within water samples adhered to the guidelines outlined in the Water and Wastewater Monitoring and Analysis Method (Table 1), as stipulated by The State Environmental Protection Administration (2002).

**TABLE 1:** Descriptive statistics for the environmental factors.

Category variables	Code	Units	Mean	Max	Min	SD
Width	Width	m	13.23	104.77	1.23	15.05
Water depth	Depth	cm	24.90	80.00	9.00	11.90
Velocity	Velocity	m/s	0.41	1.07	0.01	0.20
pH	pH	–	7.65	9.50	5.51	2.75
Water temperature	WT	°C	17.23	23.27	7.87	0.91
Conductivity	Cond	μs/cm	167.07	363.00	14.23	84.76
Permanganate index	CODMn	mg/L	1.12	2.64	0.48	0.36
Ammonia nitrogen	NH_3_–N	mg/L	0.11	0.40	0.03	0.04
Nitrate‐nitrogen	NO_3_–N	mg/L	1.02	3.40	0.21	0.55
Total nitrogen	TN	mg/L	1.80	4.20	0.81	0.60
Total phosphorus	TP	mg/L	0.05	0.51	0.01	0.05
Habitat score	QHEI	%	65.68	80.77	31.87	9.23
Elevation of the sampling site	Elevation	m	270.88	648.00	102.00	131.50
Slope of the sampling site	Slope	°	11.01	33.19	0.24	8.43
Aspect of the sampling site	Aspect	°	200.44	358.65	3.71	106.62
Forest%	Forest	%	96.05	100.00	59.79	6.62
Cropland%	Cropland	%	3.51	38.36	0.00	6.06
Shrubs%	Shrubs	%	0.01	0.25	0.00	0.03
Grassland%	Grassland	%	0.02	0.26	0.00	0.05
Water%	Water	%	0.06	2.12	0.00	0.24
Impervious%	Impervious	%	0.35	4.49	0.00	0.66

The land cover data of the sampling site at a spatial resolution of 30 m were obtained from Yang and Huang (2021). The land cover within the upstream catchment area of each sampling site was used as the land cover for that site. The study area was re‐categorized to identify six land use types: Forest, Cropland, Shrubs, Grassland, Water, and Impervious (see Table 1 and Figure S1). Topographic variables (including Elevation, Slope, and Aspect) were obtained from Amatulli et al. (2018). Slope represents the steepness of the stream along the longitudinal gradient and the aspect captures information on the north–south and east–west orientation of each location.

### 2.4 Relationships between macroinvertebrate traits and environment

In order to address the impact of dispersal on bioassessment indices, we identified the taxa most affected by dispersal by using dispersal ability group trait (DAG, Bilton et al., 2001; Heino, 2013a; Van De Meutter et al., 2007, Table 2). For detailed information on macroinvertebrates DAG traits, refer to Table S2. DAG01 represents weak passive dispersers with aquatic adults, and DAG04 refers to strong aerial dispersers with flying adults. Based on our previous study of macroinvertebrate community assembly mechanisms in TIL catchment (Liu et al., 2023), the results of trait decomposition and variance partitioning analysis showed that DAG01 and DAG04 had a total explanation of 25% and 61%. In particular, the pure effect of spatial processes on both taxa DAG01 and DAG04 (15% and 52%, respectively) accounted for 60% or more of the total explanation and was much larger than the pure effect of environmental filtering (1% and 3%, respectively). We therefore considered that the 64 species of DAG01 and DAG04 were strongly influenced by the dispersal processes.

**TABLE 2:** Macroinvertebrates dispersal ability group trait (DAG), categories, codes, and descriptions used in this study.

Trait	Codes	Categories	Descriptions
Dispersal ability	DAG01	Weak passive dispersers with aquatic adults	The classification of DAG was based on the ability of macroinvertebrates to spread on land
	DAG02	Weak aerial dispersers with flying adults	
	DAG03	Intermediate aerial dispersers with flying adults	
	DAG04	Strong aerial dispersers with flying adults	

### 2.5 Data analysis

**FIGURE 3:**
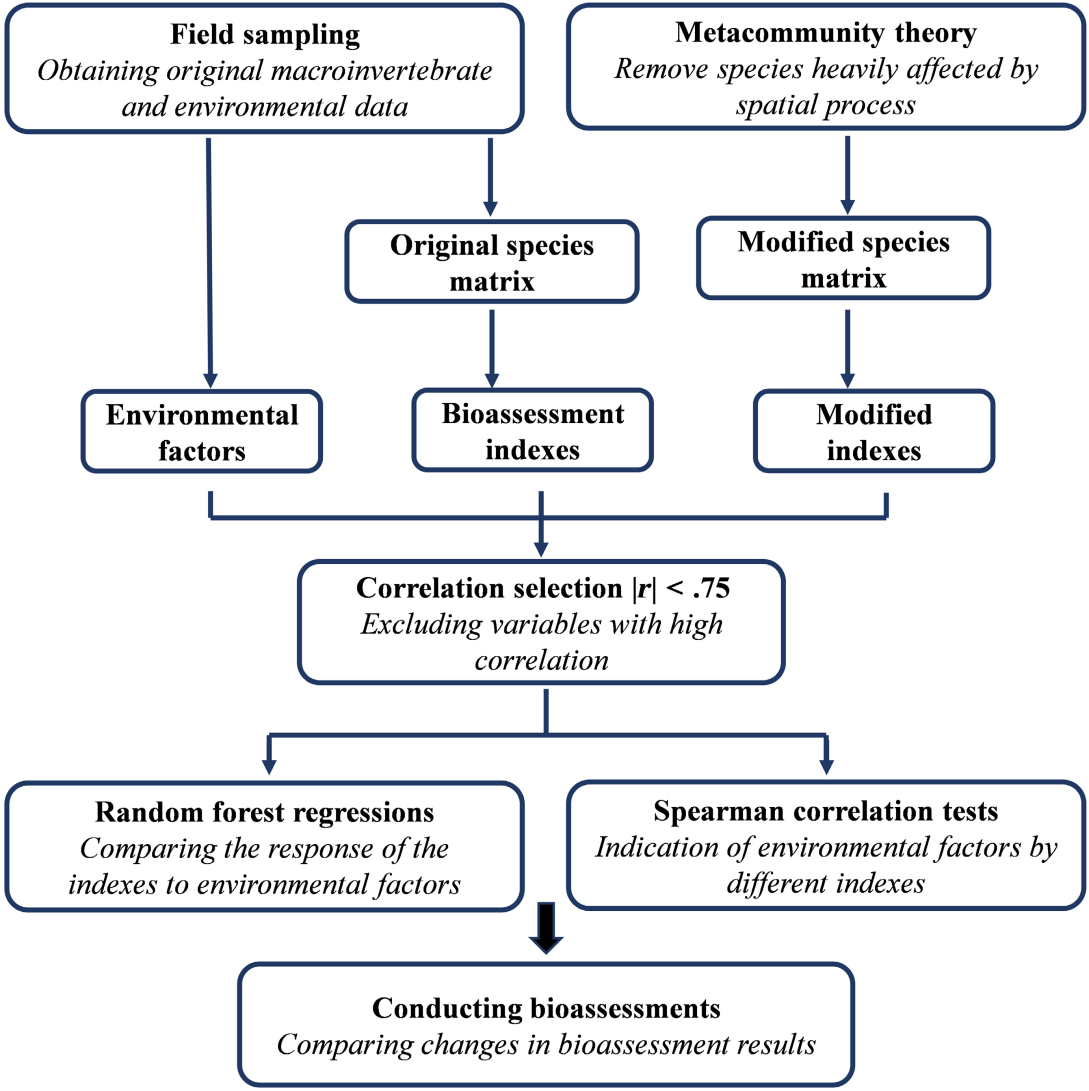
A diagram of the structure of this study.

All data processing was performed in R (Version 4.2.1, R Core Team, 2020) and SPSS (version 26, USA) (Figure 3). The original and modified indices were calculated from the original species matrix and the modified species matrix (i.e., remove DAG01 and DAG04). Following this, the H′ and $H_{\mathrm{mod}}^{\prime }$ index were calculated using the *diversity* function in package *vegan* (Oksanen et al., 2019), while the *bmwp_ind* function in the package *metrix* was employed to calculate BMWP, BMWP_mod_, ASPT, and ASPT_mod_ (Armitage et al., 1983). At the same time, we calculated the BI, BI_mod_, EPT, and EPT_mod_ based on formulas according to Kitchin (2005) and Hilsenhoff (1977):

$$\mathrm{EPT}=S_{E}+S_{P}+S_{T}$$

where *S*_*E*_, *S*_*P*_, and *S*_*T*_ are the number of taxonomic units at the family level for Ephemeroptera, Plecoptera, and Trichoptera respectively.

$$\mathrm{BI}=\frac{\sum \left(n_{i}a_{i}\right)}{N}$$

where *n* = number of specimens in taxa; *a* = tolerance value of taxa; *N* = total number of specimens in the sample.

The macroinvertebrates bioassessment indices (containing the original and modified indices) and environmental factors datasets (containing 21 variables) were used to calculate the Spearman rank correlation between the original bioassessment indices and the environmental factors by using SPSS. Prior to this, the Spearman rank correlation analysis was done separately for the indices and environmental factors, and only those with |*r*| < .75 were allowed to proceed to the next step of the analysis (Huang et al., 2015; You et al., 2021). Next, we used the *randomForest* function in the R package *randomForest* to perform random forest regression (RFR) (Breiman, 2001). RFR was used to test the relationship between each index and 21 environmental factors separately. Then, we again used the Spearman rank correlation test to calculate the correlation of environmental factors with the original and modified indices and visualized them using *pheatmap* function in R package *pheatmap* (Kolde & Kolde, 2018). Finally, we conducted a river health assessment based on the grading criteria of the bioassessment indices and used the Kruskal–Wallis tests to examine differences between overall values of each original index and index_mod_ (Table 3).

**TABLE 3:** Criteria for grading bioassessment indices.

Index	Excellent	Good	Fair	Poor	Very poor
H′	>3	(2.0, 3.0]	(1.0, 2.0]	(0.5, 1.0]	(0, 0.5]
BMWP	>80	(50, 80]	(25, 50]	(10, 25]	(0, 10]
ASPT	>4.0	(3.5, 4.0]	(3.1, 3.5]	(2.0, 3.0]	(0, 2.0]
BI	(0, 3.5]	(3.5, 5.5]	(5.5, 6.5]	(6.5, 8.5]	>8.5
EPT	>28	(20, 27]	(14, 20]	(6, 14]	(0, 6]

Abbreviations: ASPT, average score per taxon; BI, biotic index; BMWP, Biological Monitoring Working Party; EPT, EPT taxa index; H′, Shannon Weiner diversity index.

## 3 RESULTS

### 3.1 Macroinvertebrate communities and environmental conditions

In total, 199 taxa were identified, belonging to 89 families, 22 orders, 9 classes, and 4 phyla. Within this taxonomic diversity, aquatic insects made up a substantial 75% of the total richness. Particularly noteworthy were Ephemeroptera (comprising 36 taxa) and Diptera (comprising 31 taxa), both significantly abundant taxa. Additionally, we also documented 49 taxa of DAG01 and 17 taxa of DGA04 (as detailed in Table S1).

The Spearman rank correlation test for environmental factors showed that most of them were lacking in high correlation values (Table S2). The correlation between NO_3_–N and TN was high (*r* = .893). The NO_3_–N was excluded because it derived from the TN. Similarly, the correlation between Forest, Cropland, and Impervious was high (|*r*| > .75), and the forest had higher cover and variability in values, so Cropland and Impervious were excluded.

### 3.2 Correlation of bioassessment indices

The outcomes of the Spearman rank correlation analysis (see Table S3) revealed the interrelation among the five macroinvertebrate bioassessment indices (|*r*| ranging from .248 to .905, *p* < .01). This signifies a pronounced level of agreement in the capacity of these indices to gauge river health. A notably strong correlation was found between the BMWP and the EPT index (|*r*| = .905). The EPT taxa are considered to be representative of aquatic insects in clean water bodies, which may also contribute to the generally high BMWP values for these taxa. And the BMWP index was only used since there was no difference between the EPT and the modified EPT_mod_ index. The other four indices (including H′, BMWP, ASPT, and BI) did not have high correlation coefficients (|*r*| from .248 to .632), which suggested that each index responded to some independent information. Given that the BI index is derived from the tolerance values of macroinvertebrates, it exhibited a negative correlation with the other four bioassessment indices. However, this correlation was relatively weak (*r* ranging from −.408 to −.266, *p* < .01).

### 3.3 Response of bioassessment indices to environmental factors

**FIGURE 4:**
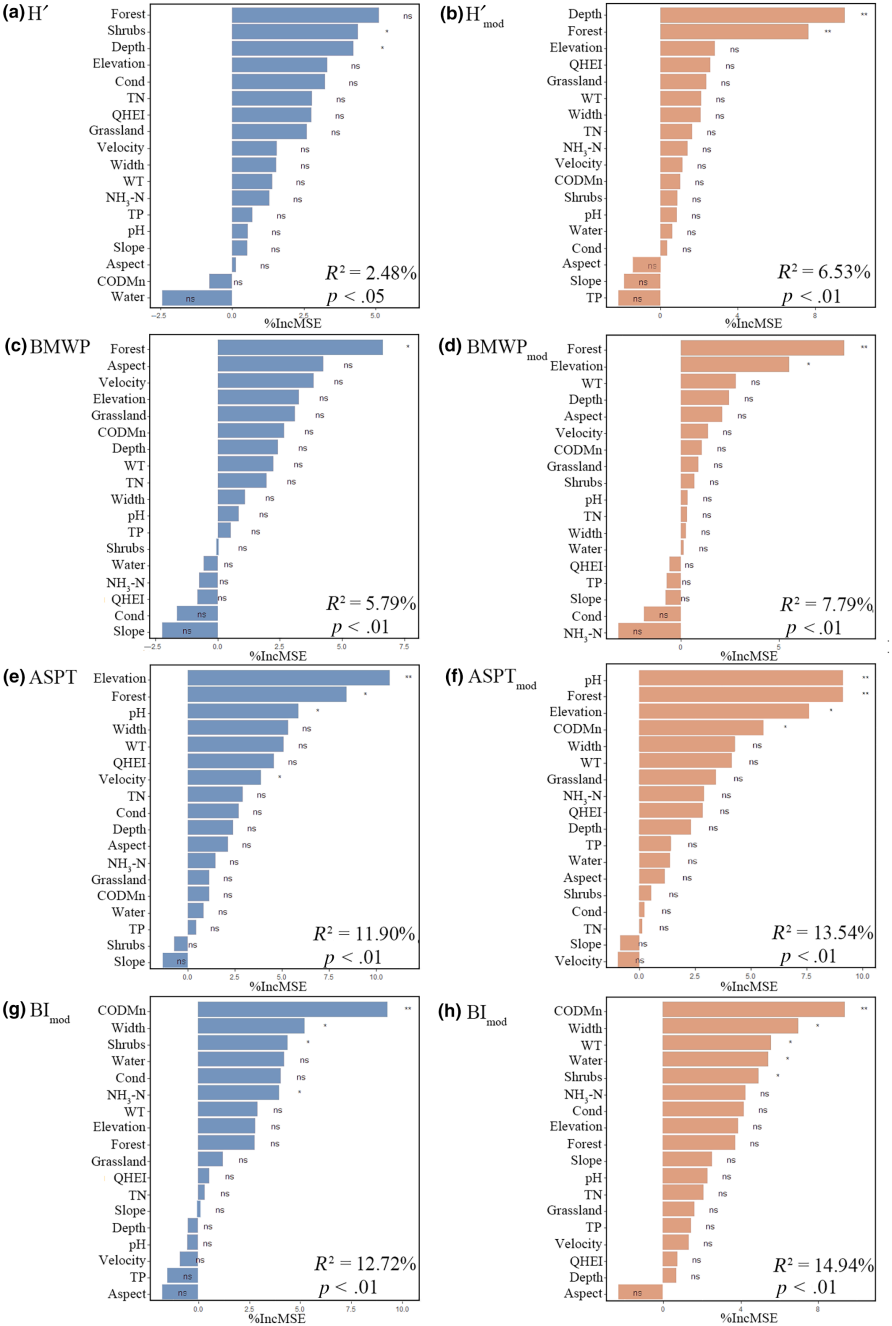
Results of random forest regressions (RFR) showing the sequence and statistical significance of environmental variables explaining macroinvertebrates (a) H′; (b) $H_{\mathrm{mod}}^{\prime }$; (c) BMWP; (d) BMWP_mod_; (e) ASPT; (f) ASPT_mod_; (g) BI; (h) BI_mod_. %IncMSE, Increased in mean squared error (%). Significance was indicated as **p < .01, *p < .05, and ns *p* ≥ .05.

The results of the RFR showed that the environmental factors explained more of the modified bioassessment indices (*R* ^2^: 6.53%–14.94%) (Figure 4a,c,e,g) than the original bioassessment indices (*R* ^2^: 2.48%–12.72%) (Figure 4b,d,f,h). Among these, the H′ index responded better with Shrubs and Depth (refer to Figure 4a), whereas the $H_{\mathrm{mod}}^{\prime }$ index exhibited Depth and Forest as the predominant predictors (as depicted in Figure 4b). A total of 27 taxa (13.6% of the total taxa) were excluded from the calculation of BMWP and ASPT according to the method of Armitage et al. (1983). For BMWP index, the best predictor was Forest (Figure 4c); the Forest and Elevation were the best predictors for the BMWP_mod_ index (Figure 4d). The ASPT index, on the other hand, revealed noteworthy responsiveness to Elevation, Forest, pH, and Velocity (as depicted in Figure 4e). Meanwhile, the ASPT_mod_ index demonstrated a strong predictive linkage with pH, Forest, Elevation, and CODMn (see Figure 4f). Turning to the BI index, its predictive power was notably associated with CODMn, Width, WT, Shrubs, and NH_3_–N (as illustrated in Figure 4g). Similarly, the BI_mod_ index displayed CODMn, Width, WT, Water, and Shrubs as its most influential predictors (depicted in Figure 4h).

**FIGURE 5:**
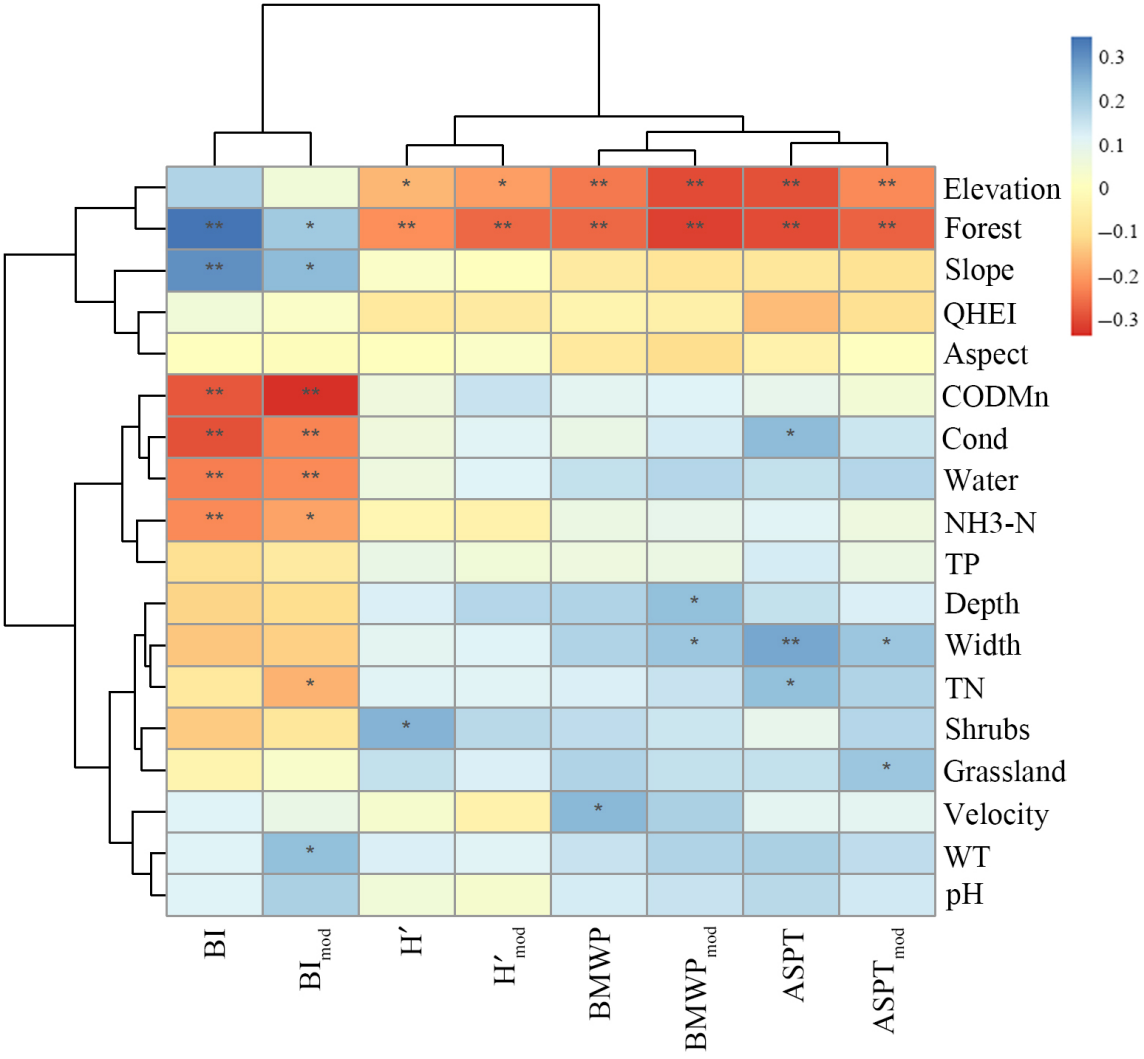
Heat map of environmental factors associated with macroinvertebrates bioassessment indices. Indices and environmental factors were clustered using hierarchical clustering and correlations were tested by Spearman correlation analysis. *R* values were differentiated by color (blue, positive; orange, negative). Significance was indicated as **p < .01, *p < .05.

The results of the correlation heat map showed that different bioassessment indices indicate different environmental factors (*R* values ranged from −0.306 to 0.334; and *p* values from 6.808E‐19 to .968) (Figure 5). Overall, almost all indices were significantly correlated with Forest. All indices except the BI and BI_mod_ indices were significantly correlated with Elevation. When examining all groups of indices, the BI and BI_mod_ indices showed the strongest correlations with environmental factors, which was consistent with the results of the RFR (Figure 4h,g), especially for water quality factors such as CODMn (*p* < .01), Cond (*p* < .01), and NH_3_–N (*p* < .01, *p* < .05, respectively). The BI_mod_ index was also significantly correlated with TN (*p* < .05) and WT (*p* < .05). H′, $H_{\mathrm{mod}}^{\prime }$, BMWP, and BMWP_mod_ indices were the least responsive to environmental factors, such that the results were also similar to those of the RFR. BMWP was significantly correlated with Velocity (*p* < .05), while BMWP_mod_ was significantly correlated with Depth (*p* < .05) and Width (*p* < .05). In addition, the ASPT and ASPT_mod_ were strongly correlated with Width (*p* < .01, *p* < .05, respectively). ASPT index was significantly correlated with Cond (*p* < .05) and TN (*p* < .05), and ASPT_mod_ was significantly correlated with Grassland (*p* < .05).

### 3.4 Differences in bioassessment results

**FIGURE 6:**
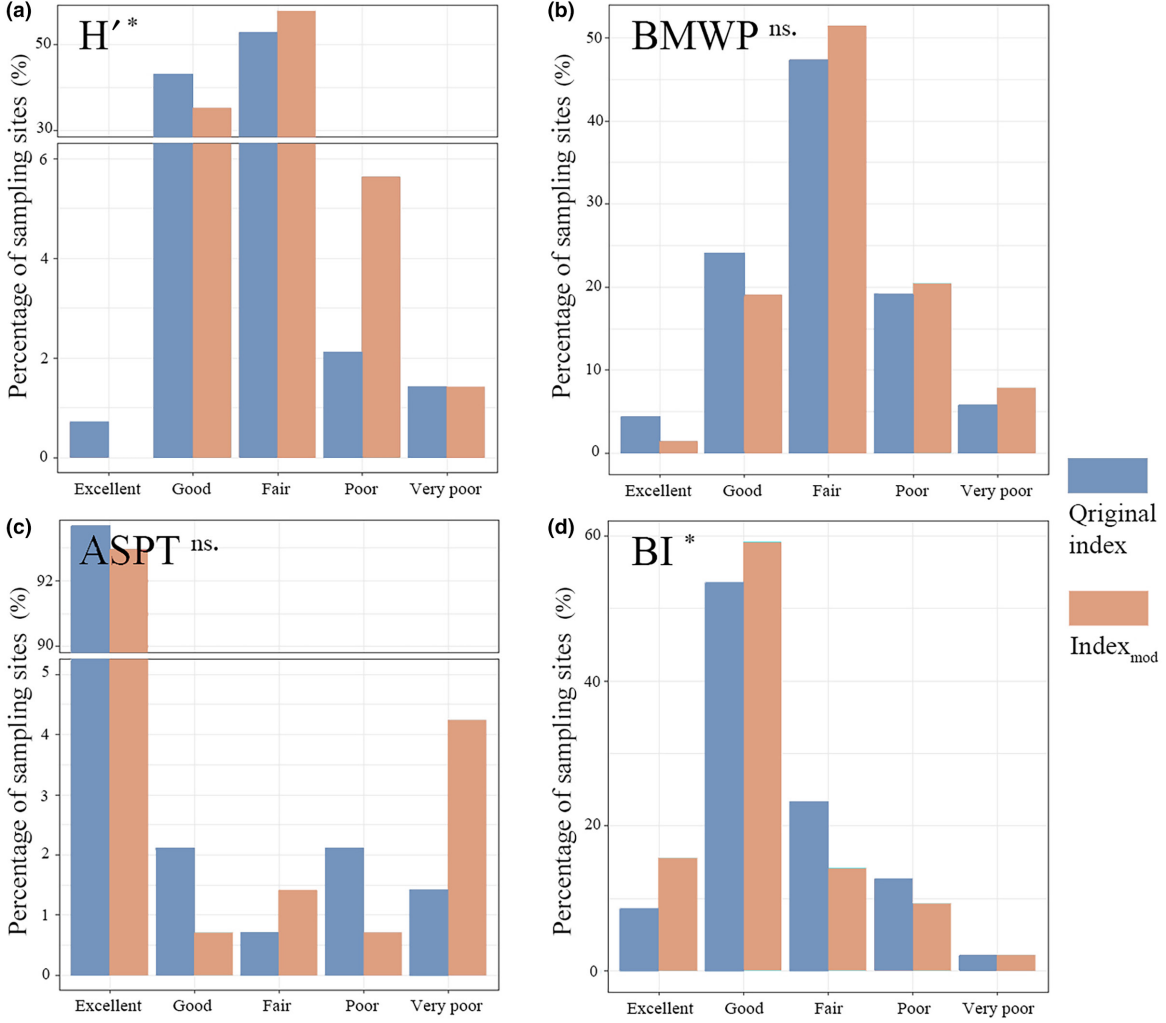
Health assessment results of different macroinvertebrates bioassessment indices for the Thousand Islands Lake (TIL) catchment. Significance was indicated as *p < .05 and ns *p* ≥ .05.

The results of Kruskal–Wallis test showed significant differences between the values of H′ and BI indices with their corresponding modified indices (i.e., *p* < .05, chi‐squared value = 4.15 and 5.40, respectively). There was no significant difference in the values of the BMWP and ASPT indices and their corresponding modified indices (i.e., *p* ≥ .05). The bioassessment results based on the different indices show that there were differences in the health assessment results of the four groups of indices, as well as differences in the bioassessment results of each index and its corresponding modified index (Figure 6). For the H′ index, 43.66% of the sampling points were excellent and good, 52.82% were fair, and 3.52% were poor and very poor, while for $H_{\mathrm{mod}}^{\prime }$ index, the percentage of excellent and good sampling points decreased (35.21%), the percentage of fair sampling points increased (57.75%), and the percentage of poor and very poor sampling points increased (7.04%) (Figure 6a). The BMWP and BMWP_mod_ showed 28.17% and 47.18% of excellent and good sampling points, 47.18% and 51.41% of fair sampling points, and 24.65% and 28.17% of poor and very poor sampling points (Figure 6b). The bioassessment results of ASPT index indicated 95.77% sampling points were excellent and good, 0.70% of fair, 3.52% were poor and very poor, compared to the ASPT_mod_ index which showed a decrease in the percentage of excellent and good sampling points (93.66%), an increase in the percentage of fair sampling points (1.41%) and an increase in the percentage of poor and very poor sampling points (4.93%) (Figure 6c). BI_mod_ showed an increase in the proportion of excellent and good sampling points (74.65%) over BI (61.97%), a decrease in the proportion of fair sampling points (23.24%–14.08%) and a decrease in the proportion of poor and very poor sampling points (14.79%–11.27%) (Figure 6d). Overall, the bioassessment results based on the $H_{\mathrm{mod}}^{\prime }$, BMWP_mod_, and ASPT_mod_ indices became worse, while the bioassessment results based on the BI_mod_ index became better.

## 4 DISCUSSION

### 4.1 Impact of spatial processes on bioassessment

Here, a novel approach is employed to explore the impact of spatial processes on bioassessment indices. Species strongly affected by spatial process, identified based on macroinvertebrate trait information (i.e., the 64 species included in DAG01 and DAG04), were excluded. This served as the basis for calculating the original and modified indices (Liu et al., 2023). The results showed the same pattern, without exception, the modified indexes were better reflections of the state of the environment than the original indexes. This indicated that consideration of spatial processes allowed for more reliable bioassessment.

Actually, advances in theoretical ecology have shown that biodiversity has a complex relationship with dispersal (Cadotte, 2006; Jiang et al., 2021). Despite recognition of the significance of dispersal in metacommunity ecology, most bioassessments are based on a “pure” species sorting view (Cai et al., 2019; Heino, 2013b; Vilmi et al., 2016). Dispersal processes may fragment the relationship between biomes and their environment (Bried & Vilmi, 2022; Leboucher et al., 2020), further potentially affecting the bioassessment. Recently, Vilmi et al. (2016) showed that species sorting was not the only factor affecting community structure. In addition, Leboucher et al. (2021) also pointed out that considering dispersal processes can improve the accuracy of biological evaluations. Indeed, the intensity of community influence by dispersal processes may depend on the dispersal capacity of organisms, spatial extent, and location in the river basin system (Armitage et al., 1983; Heino et al., 2015). Astorga et al. (2012) found that weaker dispersers exhibited lower environmental control and higher spatial structuring compared to stronger dispersers. However, Heino et al. (2012) did not find significant differences between diatoms (efficient passive dispersers), bryophytes (passive dispersers with moderate dispersal capacity), and invertebrates (inefficient active dispersers) in this regard. The difference in spatial span (distance between sites up to about 800 km and less than 100 km, respectively) between the two studies leads us to hypothesize that the degree of influence of spatial processes varies with spatial extent. The spatial extent of TIL catchment (an area of approximately 10,800 km^2^) may result in weak dispersers (i.e., DAG01) being affected by dispersal limitation. In addition, the importance of dispersal may be different at different sections of the river network. The headwater streams receive few migrants, so environmental factors should be the main influence on local communities (Schmera et al., 2018). In contrast, our sites covered not only upstream areas but also large downstream rivers connected through Thousand Island Lake, and communities at these sites may receive many migrants from tributaries or even lakes. It may be that such specialized basin configurations result in some high taxa (i.e., DAG04) potentially being affected by high dispersal rates. All these factors may distort the results of the bioassessment by affecting community dispersal. Based on these factors and previous investigation results, the novelty of our study is that we excluded species (i.e., DAG01 and DAG04) that have been identified as being more influenced by spatial processes in TIL catchment, minimizing the impact of dispersal processes on the bioassessment. According to our results, the relationship between the modified indices and the environment was higher than those of the original indices, which led us to conclude that the modified indices might be better indicators of environmental conditions. Thus, the impact of spatial processes should be emphasized in future research to allow for more reliable bioassessments.

### 4.2 Relationship between bioassessment indices and environmental factors

Our results show that different bioassessment indices were correlated with different environmental factors (Figure 5). Specifically, the BMWP and ASPT and their corresponding modified indices reflected changes in hydro‐morphological factors such as width, but poorly reflect water quality factors. As shown by Kantzaris et al. (2002), BMWP and ASPT were not sufficient to assess water quality. But previous studies have also found the applicability of BMWP and ASPT to reflect the physical and chemical factors of water (Yorulmaz & Ertas, 2021; Zeybek, 2017). We speculate that this might be due to the similarity of water quality factors across TIL catchment. BMWP and ASPT were used at the family level. In parallel, family‐level values usually represent an intermediate value of species sensitivity (Armitage et al., 1983). In addition, another possible reason is that the BMWP and ASPT indices constructed on the basis of the biological status of UK rivers may not be fully applicable to the TIL. Compared to species‐level‐based indices, family‐level indices may underestimate or overestimate water quality and therefore do not accurately reflect changes in water quality in our study area (MacNeil & Briffa, 2009). Meanwhile, the strongest relationships were found between the BI and BI_mod_ indices and environmental factors, especially with water quality factors such as CODMn, Cond, and NH_3_–N. The accuracy of the BI index in reflecting water quality factors had also been noted in previous studies (Wang et al., 2021; Zhang et al., 2020).

In addition, the H′ and $H_{\mathrm{mod}}^{\prime }$ appeared to respond only to changes in Elevation and Forest. The results also showed that all indices except BI and BI_mod_ were significantly correlated with Elevation. Recent studies have found that macroinvertebrates diversity patterns are influenced by elevation, particularly in montane stream ecosystems, while altitude may also indirectly affect macroinvertebrates abundance by causing changes in gradients in environmental factors such as climate (Chiu et al., 2020; Yang et al., 2020). These changes may further influence bioassessment. Moreover, all indices were significantly correlated with Forest, and changes in Forest may have an elevational gradient. Land use change (e.g., natural vegetation being replaced by anthropogenic land use) can degrade riparian zones and stream habitats in different ways (Siqueira et al., 2015; Stendera et al., 2012). Therefore, land use can significantly alter the biodiversity of freshwater (Carlson et al., 2016; Johnson et al., 2013). Our study area is a well‐protected, highly forested watershed of great ecological significance. Hence, it is important to protect its ecological integrity (Fu et al., 2022; Sheng & Han, 2022a).

### 4.3 Applicability and comparison of bioassessment indices

In this study, differences in the bioassessment results based on the original and modified indices were found (Figure 6). Overall, the assessment results showed that the steam health in TIL catchment was relatively good. This may be due to the fact that this region is generally subject to less human disturbance, which may result in inadequate coverage of all disturbance gradients. Future studies should thus be expanded to other catchments with significant anthropogenic gradients and assess the generalizability of our findings. Specifically, the original H′, BMWP, and ASPT indices overestimated the bioassessment results, in contrast to the original BI index which underestimated the bioassessment results. Based on our results, the relationship between the modified index and the environment is higher than that of the original index. Therefore, bioassessments based on the modified indices may be more reliable. But the differences between the indices and their applicability in the region will be the focus of our discussion below.

Specifically, traditional biodiversity indices, such as the H′ index, have proved useful in assessing the quality of aquatic ecosystems (Heino et al., 2007; Ji et al., 2020). However, from the results of RFR, it appeared that neither the H′ nor the modified $H_{\mathrm{mod}}^{\prime }$ were good indicators of environmental change. At the same time, the accuracy of these indices is increasingly questioned because they ignore functional and phylogenetic differences between species (Jiang et al., 2014; Leonard et al., 2006). Therefore, traditional biodiversity indices alone cannot be relied upon for bioassessment of this region. The BMWP index and the improved ASPT index take into account the sensitivity values of taxonomic units at the family level and are less demanding for species classification, but they tend to be highly variable across regions and the sensitivity values of macroinvertebrates within the same family tend to vary considerably (Monaghan & Soares, 2012; Varnosfaderany et al., 2010). From the RFR results, the BMWP and BMWP_mod_ are poor indicators of environmental factors. A previous study by Ogleni and Topal (2011) also pointed out the inadequacy of the BMWP for water quality assessment, while the ASPT and ASPT_mod_ were more indicative of environmental factors. Varnosfaderany et al. (2010) also showed that the ASPT was more responsive to environmental factors than the BMWP. The BI index, on the other hand, considers the tolerance values of the species and places increased demands on species classification (Carter et al., 2007; Pawlowski et al., 2018). Meanwhile, the strongest relationships were found between the BI and BI_mod_ indices and environmental factors. The ASPT and BI and their corresponding modified indices were thus more applicable to the bioassessment of the TIL catchment. However, domestic researches (China) on tolerance and sensitivity values are still very limited, making it necessary to refer to foreign standards for the application of indices, so future efforts should be made in this direction to target the development of tolerance and sensitivity values and that are consistent with specific regions (Liu et al., 2019; Zhang et al., 2020).

At the same time, there is a significant difference between the original and modified indices (here especially H′ and BI indices). Such results once again demonstrate the impact of dispersal processes on bioassessment. Previous studies have combined spatial and trait analyses to measure the importance of spatial processes (dispersal limitation and mass effects) in the community structure of different dispersers. The present study demonstrates the significant impact of spatial processes on bioassessment indices (especially H′ and BI indices). Leboucher et al. (2021) identified 40 diatom species in France with distributions driven primarily by mass effects and proposed an index of biological diatoms (IBD_mod_) that excludes these species. By comparing the differences between the original IBD and IBD_mod_ responses to the environment, it was also demonstrated that mass effects may lead to biased water quality evaluations. Our approach is novel and easy to use compared to passive dispersers such as diatoms, but it is possible that the effects of spatial processes cannot be accurately quantified due to the coarse levels associated with DAG. But there is no doubt that spatial processes affect bioassessment in both active and passive dispersers. Therefore, the accuracy and validity of the original bioassessment needs to be re‐examined in the context of metacommunity theory and the idea can be applied to other highly connected freshwater ecosystems (Cai et al., 2019; Leboucher et al., 2021; Vilmi et al., 2016).

## 5 CONCLUSION

In conclusion, our study has revealed several key insights into the realm of macroinvertebrate bioassessment indices and their applicability. Firstly, we established that distinct bioassessment indices exhibited significant correlations, yet their responses to environmental factors varied considerably. Notably, the modified indices showcased differential preferences in reflecting environmental nuances, highlighting the importance of their careful selection based on study objectives and contexts. Secondly, the modified bioassessment indices emerged as more robust indicators of environmental conditions compared to their original counterparts, emphasizing the role of spatial processes in refining bioassessment accuracy. However, the degree of accuracy of specific indices needs to be explored in different types of regions. Lastly, a noteworthy disparity in bioassessment outcomes was observed between the modified and original indices, underscoring the need for a comprehensive understanding of spatial processes' influence on these outcomes.

Freshwater ecosystems are irreplaceable for human survival and social development, while multiple stressors threaten them. Our study confirms that existing traditional bioassessment results based on species‐environment relationships deviate from the actual freshwater ecosystem health. Therefore, there is an urgent need to apply metacommunity theory to bioassessments to reduce the influence of biological dispersal processes on freshwater ecosystem's health assessment results. Finally, we suggest that bioassessment considering metacommunity theory can be applied to other aquatic communities (e.g., fish, algae, and phytoplankton) and different freshwater ecosystems (e.g., lakes, reservoirs and wetlands). In light of our study's contributions, we contend that the advancement of bioassessment methodologies through metacommunity theory consideration holds promise for more accurate and reliable freshwater ecosystem health evaluations. This, in turn, could facilitate informed decision‐making in conservation and management efforts, ensuring the preservation of these invaluable ecosystems for future generations. As the ecological intricacies of freshwater environments continue to be unraveled, the adoption of innovative approaches like metacommunity theory‐inspired bioassessment could pave the way toward a more sustainable coexistence between human society and aquatic ecosystems.

## AUTHOR CONTRIBUTIONS

**Guohao Liu:** Conceptualization (equal); investigation (equal); writing – original draft (equal). **Xinxin Qi:** Data curation (equal); investigation (equal). **Zongwei Lin:** Data curation (equal); investigation (equal). **Yuanyuan Lv:** Data curation (equal); investigation (equal). **Sangar Khan:** Supervision (equal); writing – review and editing (equal). **Xiaodong Qu:** Conceptualization (equal); methodology (equal). **Binsong Jin:** Methodology (equal); supervision (equal). **Ming Wu:** Methodology (equal); supervision (equal). **Collins Oduro:** Writing – review and editing (equal). **Naicheng Wu:** Conceptualization (equal); funding acquisition (equal); investigation (equal); project administration (equal); writing – review and editing (equal).

## CONFLICT OF INTEREST STATEMENT

The authors declare that they have no known competing financial interests or personal relationships that could have appeared to influence the work reported in this paper.

## Supporting information


Appendix S1:

## Data Availability

All data are uploaded as supporting material.

## References

[ece310896-bib-0001] Albert, J. S. , Destouni, G. , Duke‐Sylvester, S. M. , Magurran, A. E. , Oberdorff, T. , Reis, R. E. , Winemiller, K. O. , & Ripple, W. J. (2021). Scientists' warning to humanity on the freshwater biodiversity crisis. Ambio, 50(1), 85–94. 10.1007/s13280-020-01318-8 32040746 PMC7708569

[ece310896-bib-0002] Amatulli, G. , Domisch, S. , Tuanmu, M.‐N. , Parmentier, B. , Ranipeta, A. , Malczyk, J. , & Jetz, W. (2018). A suite of global, cross‐scale topographic variables for environmental and biodiversity modeling. Scientific Data, 5(1), 180040. 10.1038/sdata.2018.40 29557978 PMC5859920

[ece310896-bib-0003] Angeler, D. G. , Allen, C. R. , Birge, H. E. , Drakare, S. , McKie, B. G. , & Johnson, R. K. (2014). Assessing and managing freshwater ecosystems vulnerable to environmental change. Ambio, 43, 113–129. 10.1007/s13280-014-0566-z 25403974 PMC4235931

[ece310896-bib-0004] Arle, J. , Mohaupt, V. , & Kirst, I. (2016). Monitoring of surface waters in Germany under the water framework directive‐a review of approaches, methods and results. Water, 8(6), 217. 10.3390/w8060217

[ece310896-bib-0005] Armitage, P. D. , Moss, D. , Wright, J. F. , & Furse, M. T. (1983). The performance of a new biological water quality score system based on macroinvertebrates over a wide range of unpolluted running‐water sites. Water Research, 17(3), 333–347. 10.1016/0043-1354(83)90188-4

[ece310896-bib-0006] Astorga, A. , Oksanen, J. , Luoto, M. , Soininen, J. , Virtanen, R. , & Muotka, T. (2012). Distance decay of similarity in freshwater communities: Do macro‐ and microorganisms follow the same rules? Global Ecology and Biogeography, 21(3), 365–375. 10.1111/j.1466-8238.2011.00681.x

[ece310896-bib-0007] Bilton, D. T. , Freeland, J. R. , & Okamura, B. (2001). Dispersal in freshwater invertebrates. Annual Review of Ecology and Systematics, 32, 159–181. 10.1146/annurev.ecolsys.32.081501.114016

[ece310896-bib-0008] Breiman, L. (2001). Random forests. Machine Learning, 45, 5–32. 10.1023/A:1010933404324

[ece310896-bib-0009] Bried, J. T. , & Vilmi, A. (2022). Improved detection of mass effect species assembly for applied metacommunity thinking. Journal of Applied Ecology, 59(4), 921–926. 10.1111/1365-2664.14115

[ece310896-bib-0010] Buss, D. F. , Carlisle, D. M. , Chon, T.‐S. , Culp, J. , Harding, J. S. , Keizer‐Vlek, H. E. , Robinson, W. A. , Strachan, S. , Thirion, C. , & Hughes, R. M. (2015). Stream biomonitoring using macroinvertebrates around the globe: A comparison of large‐scale programs. Environmental Monitoring and Assessment, 187(1), 4132. 10.1007/s10661-014-4132-8 25487459

[ece310896-bib-0011] Cadotte, M. W. (2006). Dispersal and species diversity: A meta‐analysis. American Naturalist, 167(6), 913–924. 10.1086/504850 16649154

[ece310896-bib-0012] Cai, Y. , Zhang, Y. , Hu, Z. , Deng, J. , Qin, B. , Yin, H. , Wang, X. , Gong, Z. , & Heino, J. (2019). Metacommunity ecology meets bioassessment: Assessing spatio‐temporal variation in multiple facets of macroinvertebrate diversity in human‐influenced large lakes. Ecological Indicators, 103, 713–721. 10.1016/j.ecolind.2019.03.016

[ece310896-bib-0013] Carlson, P. E. , McKie, B. G. , Sandin, L. , & Johnson, R. K. (2016). Strong land‐use effects on the dispersal patterns of adult stream insects: Implications for transfers of aquatic subsidies to terrestrial consumers. Freshwater Biology, 61(6), 848–861. 10.1111/fwb.12745

[ece310896-bib-0014] Carpenter, S. R. , Stanley, E. H. , & Vander Zanden, M. J. (2011). State of the World's freshwater ecosystems: Physical, chemical, and biological changes. Annual Review of Environment and Resources, 36(36), 75–99. 10.1146/annurev-environ-021810-094524

[ece310896-bib-0015] Carter, J. L. , Resh, V. H. , Hannaford, M. J. , & Myers, M. J. (2007). CHAPTER 35 – macroinvertebrates as biotic indicators of environmental quality. In F. R. Hauer & G. A. Lamberti (Eds.), Methods in stream ecology (2nd ed., pp. 805–831). Academic Press.

[ece310896-bib-0016] Chiu, M.‐C. , Ao, S. , He, F. , Resh, V. H. , & Cai, Q. (2020). Elevation shapes biodiversity patterns through metacommunity‐structuring processes. Science of the Total Environment, 743, 140548. 10.1016/j.scitotenv.2020.140548 32758813

[ece310896-bib-0017] Cottenie, K. (2005). Integrating environmental and spatial processes in ecological community dynamics. Ecology Letters, 8(11), 1175–1182. 10.1111/j.1461-0248.2005.00820.x 21352441

[ece310896-bib-0018] De Bie, T. , De Meester, L. , Brendonck, L. , Martens, K. , Goddeeris, B. , Ercken, D. , Hampel, H. , Denys, L. , Vanhecke, L. , Van der Gucht, K. , Van Wichelen, J. , Vyverman, W. , & Declerck, S. A. J. (2012). Body size and dispersal mode as key traits determining metacommunity structure of aquatic organisms. Ecology Letters, 15(7), 740–747. 10.1111/j.1461-0248.2012.01794.x 22583795

[ece310896-bib-0019] Feld, C. K. , & Hering, D. (2007). Community structure or function: Effects of environmental stress on benthic macroinvertebrates at different spatial scales. Freshwater Biology, 52(7), 1380–1399. 10.1111/j.1365-2427.2007.01749.x

[ece310896-bib-0020] Fu, L. , Ren, Y. , Lu, L. , & Chen, H. (2022). Relationship between ecosystem services and rural residential well‐being in the Xin?An river basin, China. Ecological Indicators, 140, 108997. 10.1016/j.ecolind.2022.108997

[ece310896-bib-0021] Grill, G. , Lehner, B. , Thieme, M. , Geenen, B. , Tickner, D. , Antonelli, F. , Babu, S. , Borrelli, P. , Cheng, L. , Crochetiere, H. , Ehalt Macedo, H. , Filgueiras, R. , Goichot, M. , Higgins, J. , Hogan, Z. , Lip, B. , McClain, M. E. , Meng, J. , Mulligan, M. , … Zarfl, C. (2019). Mapping the world's free‐flowing rivers. Nature, 569(7755), 215–221. 10.1038/s41586-019-1111-9 31068722

[ece310896-bib-0022] Gu, Q. , Zhang, Y. , Ma, L. G. , Li, J. D. , Wang, K. , Zheng, K. F. , Zhang, X. , & Sheng, L. (2016). Assessment of reservoir water quality using multivariate statistical techniques: A case study of Qiandao Lake, China. Sustainability, 8(3), 243. 10.3390/su8030243

[ece310896-bib-0023] Heino, J. (2013a). Does dispersal ability affect the relative importance of environmental control and spatial structuring of littoral macroinvertebrate communities? Oecologia, 171(4), 971–980. 10.1007/s00442-012-2451-4 22961400

[ece310896-bib-0024] Heino, J. (2013b). The importance of metacommunity ecology for environmental assessment research in the freshwater realm. Biological Reviews, 88(1), 166–178. 10.1111/j.1469-185X.2012.00244.x 22937892

[ece310896-bib-0025] Heino, J. , Alahuhta, J. , Ala‐Hulkko, T. , Antikainen, H. , Bini, L. M. , Bonada, N. , Datry, T. , Erős, T. , Hjort, J. , Kotavaara, O. , Melo, A. S. , & Soininen, J. (2017). Integrating dispersal proxies in ecological and environmental research in the freshwater realm. Environmental Reviews, 25(3), 334–349. 10.1139/er-2016-0110

[ece310896-bib-0026] Heino, J. , Grönroos, M. , Soininen, J. , Virtanen, R. , & Muotka, T. (2012). Context dependency and metacommunity structuring in boreal headwater streams. Oikos, 121(4), 537–544. 10.1111/j.1600-0706.2011.19715.x

[ece310896-bib-0027] Heino, J. , Melo, A. S. , Siqueira, T. , Soininen, J. , Valanko, S. , & Bini, L. M. (2015). Metacommunity organisation, spatial extent and dispersal in aquatic systems: Patterns, processes and prospects. Freshwater Biology, 60(5), 845–869. 10.1111/fwb.12533

[ece310896-bib-0028] Heino, J. , Mykra, H. , Hamalainen, H. , Aroviita, J. , & Muotka, T. (2007). Responses of taxonomic distinctness and species diversity indices to anthropogenic impacts and natural environmental gradients in stream macroinvertebrates. Freshwater Biology, 52(9), 1846–1861. 10.1111/j.1365-2427.2007.01801.x

[ece310896-bib-0029] Hering, D. , Johnson, R. K. , Kramm, S. , Schmutz, S. , Szoszkiewicz, K. , & Verdonschot, P. F. M. (2006). Assessment of European streams with diatoms, macrophytes, macroinvertebrates and fish: A comparative metric‐based analysis of organism response to stress. Freshwater Biology, 51(9), 1757–1785. 10.1111/j.1365-2427.2006.01610.x

[ece310896-bib-0030] Hilsenhoff, W. L. (1977). Use of arthropods to evaluate water quality of streams [Wisconsin]. Technical Bulletin‐Wisconsin Dept. of Natural Resources, Division of Conservation (USA).

[ece310896-bib-0031] Holzenthal, R. W. (2009). Trichoptera (caddisflies). In G. E. Likens (Ed.), Encyclopedia of inland waters (pp. 456–467). Elsevier Inc.

[ece310896-bib-0032] Huang, Q. , Gao, J. , Cai, Y. , Yin, H. , Gao, Y. , Zhao, J. , Liu, L. , & Huang, J. (2015). Development and application of benthic macroinvertebrate‐based multimetric indices for the assessment of streams and rivers in the Taihu Basin, China. Ecological Indicators, 48, 649–659. 10.1016/j.ecolind.2014.09.014

[ece310896-bib-0033] Ji, L. , Jiang, X. , Liu, C. , Xu, Z. , Wang, J. , Qian, S. , & Zhou, H. (2020). Response of traditional and taxonomic distinctness diversity indices of benthic macroinvertebrates to environmental degradation gradient in a large Chinese shallow lake. Environmental Science and Pollution Research, 27(17), 21804–21815. 10.1007/s11356-020-08610-w 32281066

[ece310896-bib-0034] Jiang, X. , Pan, B. , Jiang, W. , Hou, Y. , Yang, H. , Zhu, P. , & Heino, J. (2021). The role of environmental conditions, climatic factors and spatial processes in driving multiple facets of stream macroinvertebrate beta diversity in a climatically heterogeneous mountain region. Ecological Indicators, 124, 107407. 10.1016/j.ecolind.2021.107407

[ece310896-bib-0035] Jiang, X. , Song, Z. , Xiong, J. , & Xie, Z. (2014). Can excluding non‐insect taxa from stream macroinvertebrate surveys enhance the sensitivity of taxonomic distinctness indices to human disturbance? Ecological Indicators, 41, 175–182. 10.1016/j.ecolind.2014.01.036

[ece310896-bib-0036] Johnson, P. T. J. , Hoverman, J. T. , McKenzie, V. J. , Blaustein, A. R. , & Richgels, K. L. D. (2013). Urbanization and wetland communities: Applying metacommunity theory to understand the local and landscape effects. Journal of Applied Ecology, 50(1), 34–42. 10.1111/1365-2664.12022

[ece310896-bib-0037] Kantzaris, V. , Iliopoulou‐Georgudaki, J. , Katharios, P. , & Kaspiris, P. (2002). A comparison of several biotic indices used for water quality assessment at the Greek rivers. Fresenius Environmental Bulletin, 11(11), 1000–1007.

[ece310896-bib-0038] Kitchin, P. L. (2005). Measuring the amount of statistical information in the EPT index. Environmetrics, 16(1), 51–59. 10.1002/env.670

[ece310896-bib-0039] Kolde, R. , & Kolde, M. R. (2018). Package ‘pheatmap’ . R Package 1(10).

[ece310896-bib-0040] Kundzewicz, Z. W. , Mata, L. J. , Arnell, N. W. , Doell, P. , Jimenez, B. , Miller, K. , Oki, T. , Şen, Z. , & Shiklomanov, I. (2008). The implications of projected climate change for freshwater resources and their management. Hydrological Sciences Journal‐Journal des Sciences Hydrologiques, 53(1), 3–10. 10.1623/hysj.53.1.3

[ece310896-bib-0041] Leboucher, T. , Mignien, L. , Wach, M. , Boutry, S. , Jamoneau, A. , Passy, S. I. , & Tison‐Rosebery, J. (2021). Consideration of mass effect processes in bioindication allows more accurate bioassessment of water quality. Ecological Indicators, 127, 107791. 10.1016/j.ecolind.2021.107791

[ece310896-bib-0042] Leboucher, T. , Tison‐Rosebery, J. , Budnick, W. , Jamoneau, A. , Vyverman, W. , Soininen, J. , Boutry, S. , & Passy, S. I. (2020). A metacommunity approach for detecting species influenced by mass effect. Journal of Applied Ecology, 57(10), 2031–2040. 10.1111/1365-2664.13701

[ece310896-bib-0043] Leibold, M. A. , Holyoak, M. , Mouquet, N. , Amarasekare, P. , Chase, J. M. , Hoopes, M. F. , Holt, R. D. , Shurin, J. B. , Law, R. , Tilman, D. , Loreau, M. , & Gonzalez, A. (2004). The metacommunity concept: A framework for multi‐scale community ecology. Ecology Letters, 7(7), 601–613. 10.1111/j.1461-0248.2004.00608.x

[ece310896-bib-0044] Leonard, D. R. P. , Clarke, K. R. , Somerfield, P. J. , & Warwick, R. M. (2006). The application of an indicator based on taxonomic distinctness for UK marine biodiversity assessments. Journal of Environmental Management, 78(1), 52–62. 10.1016/j.jenvman.2005.04.008 16095807

[ece310896-bib-0045] Li, Z. , Heino, J. , Chen, X. , Liu, Z. , Meng, X. , Jiang, X. , Ge, Y. , Chen, J. , & Xie, Z. (2021). Understanding macroinvertebrate metacommunity organization using a nested study design across a mountainous river network. Ecological Indicators, 121, 107188. 10.1016/j.ecolind.2020.107188

[ece310896-bib-0092] Lin, Z. , Liu, G. , Guo, K. , Wang, K. , Wijewardene, L. , & Wu, N. (2024). Scales matter: Regional environment factors affect α diversity but local factors affect β diversity of macroinvertebrates in Thousand Islands Lake catchment area. Ecological Indicators, 158, 111561. 10.1016/j.ecolind.2024.111561

[ece310896-bib-0046] Linares, M. S. , Callisto, M. , & Marques, J. C. (2020). Assessing biological diversity and thermodynamic indicators in the dam decommissioning process. Ecological Indicators, 109, 105832. 10.1016/j.ecolind.2019.105832

[ece310896-bib-0047] Lindholm, M. , Gronroos, M. , Hjort, J. , Karjalainen, S. M. , Tokola, L. , & Heino, J. (2018). Different species trait groups of stream diatoms show divergent responses to spatial and environmental factors in a subarctic drainage basin. Hydrobiologia, 816(1), 213–230. 10.1007/s10750-018-3585-0

[ece310896-bib-0048] Liu, G. , Lin, Z. , Qi, X. , Wang, Y. , Wang, Y. , Jiang, W. , He, F. , & Wu, N. (2023). Environmental filtering, spatial processes and biotic interactions jointly shape different traits communities of stream macroinvertebrates. Frontiers in Ecology and Evolution, 11, 1196296. 10.3389/fevo.2023.1196296

[ece310896-bib-0049] Liu, W. , Xu, M. , Zhao, N. , Zhou, X. , Pan, B. , Tian, S. , & Lei, F. (2019). River health assessment of the Yellow River source region, Qinghai‐Tibetan plateau, China, based on tolerance values of macroinvertebrates. Environmental Science and Pollution Research, 26(10), 10251–10262. 10.1007/s11356-018-04110-0 30761487

[ece310896-bib-0050] Luo, K. , Hu, X. , He, Q. , Wu, Z. , Cheng, H. , Hu, Z. , & Mazumder, A. (2018). Impacts of rapid urbanization on the water quality and macroinvertebrate communities of streams: A case study in Liangjiang new area, China. Science of the Total Environment, 621, 1601–1614. 10.1016/j.scitotenv.2017.10.068 29054671

[ece310896-bib-0051] MacNeil, C. , & Briffa, M. (2009). Replacement of a native freshwater macroinvertebrate species by an invader: Implications for biological water quality monitoring. Hydrobiologia, 635(1), 321–327. 10.1007/s10750-009-9924-4

[ece310896-bib-0052] Mereta, S. T. , Boets, P. , De Meester, L. , & Goethals, P. L. M. (2013). Development of a multimetric index based on benthic macroinvertebrates for the assessment of natural wetlands in Southwest Ethiopia. Ecological Indicators, 29, 510–521. 10.1016/j.ecolind.2013.01.026

[ece310896-bib-0053] Merritt, R. W. , Cummins, K. W. , & Berg, M. B. (2017). Trophic relationships of macroinvertebrates. In F. R. Hauer & G. A. Lamberti (Eds.), Methods in stream ecology, volume 1 (pp. 413–433). Elsevier.

[ece310896-bib-0054] Monaghan, K. A. , & Soares, A. M. V. M. (2012). Bringing new knowledge to an old problem: Building a biotic index from lotic macroinvertebrate traits. Ecological Indicators, 20, 213–220. 10.1016/j.ecolind.2012.02.017

[ece310896-bib-0055] Morse, J. C. , Lianfang, Y. , & Lixin, T. (1994). Aquatic insects of China useful for monitoring water quality. Hohai University Press.

[ece310896-bib-0056] Moss, B. (2008). Water pollution by agriculture. Philosophical Transactions of the Royal Society, B: Biological Sciences, 363(1491), 659–666. 10.1098/rstb.2007.2176 PMC2610176 17666391

[ece310896-bib-0057] Mouquet, N. , & Loreau, M. (2002). Coexistence in Metacommunities: The regional similarity hypothesis. The American Naturalist, 159(4), 420–426. 10.1086/338996 18707425

[ece310896-bib-0058] Mouquet, N. , Munguia, P. , Kneitel, J. M. , & Miller, T. E. (2003). Community assembly time and the relationship between local and regional species richness. Oikos, 103(3), 618–626. 10.1034/j.1600-0706.2003.12772.x

[ece310896-bib-0059] Ogleni, N. , & Topal, B. (2011). Water quality assessment of the Mudurnu River, Turkey, using biotic indices. Water Resources Management, 25(10), 2487–2508. 10.1007/s11269-011-9822-1

[ece310896-bib-0060] Oksanen, J. , Blanchet, F. G. , Friendly, M. , Kindt, R. , & Wagner, H. H. (2019). Vegan community ecology package version 2.5‐6 .

[ece310896-bib-0061] Pawlowski, J. , Kelly‐Quinn, M. , Altermatt, F. , Apotheloz‐Perret‐Gentil, L. , Beja, P. , Boggero, A. , Borja, A. , Bouchez, A. , Cordier, T. , & Domaizon, I. (2018). The future of biotic indices in the ecogenomic era: Integrating (e) DNA metabarcoding in biological assessment of aquatic ecosystems. Science of the Total Environment, 637, 1295–1310. 10.1016/j.scitotenv.2018.05.002 29801222

[ece310896-bib-0062] R Core Team . (2020). R: A language and environment for statistical computing. R Foundation for Statistical Computing.

[ece310896-bib-0063] Ruse, L. P. (1996). Multivariate techniques relating macroinvertebrate and environmental data from a river catchment. Water Research, 30(12), 3017–3024. 10.1016/s0043-1354(96)00217-5

[ece310896-bib-0064] Schmera, D. , Árva, D. , Boda, P. , Bódis, E. , Bolgovics, Á. , Borics, G. , Csercsa, A. , Deák, C. , Krasznai, E. Á. , Lukács, B. A. , Mauchart, P. , Móra, A. , Sály, P. , Specziár, A. , Süveges, K. , Szivák, I. , Takács, P. , Tóth, M. , Várbíró, G. , … Erős, T. (2018). Does isolation influence the relative role of environmental and dispersal‐related processes in stream networks? An empirical test of the network position hypothesis using multiple taxa. Freshwater Biology, 63(1), 74–85. 10.1111/fwb.12973

[ece310896-bib-0065] Sheng, J. , & Han, X. (2022a). Practicing policy mobility of payment for ecosystem services through assemblage and performativity: Lessons from China's Xin'an River Basin eco‐compensation pilot. Ecological Economics, 191, 107234. 10.1016/j.ecolecon.2021.107234

[ece310896-bib-0066] Sheng, J. , & Han, X. (2022b). State rescaling, power reconfiguration and path dependence: China's Xin'an River Basin eco‐compensation pilot (XRBEP). Regional Studies, 56(11), 1814–1828. 10.1080/00343404.2021.2009454

[ece310896-bib-0067] Siqueira, T. , Durães, L. D. , & de Oliveira Roque, F. (2014). Predictive modelling of insect Metacommunities in biomonitoring of aquatic networks. In C. Ferreira & W. Godoy (Eds.), Ecological modelling applied to entomology (pp. 109–126). Springer International Publishing. 10.1007/978-3-319-06877-0_5

[ece310896-bib-0068] Siqueira, T. , Teixeira Lacerda, C. G.‐L. , & Saito, V. S. (2015). How does landscape modification induce biological homogenization in tropical stream Metacommunities? Biotropica, 47(4), 509–516. 10.1111/btp.12224

[ece310896-bib-0069] Stendera, S. , Adrian, R. , Bonada, N. , Canedo‐Argueelles, M. , Hugueny, B. , Januschke, K. , Pletterbauer, F. , & Hering, D. (2012). Drivers and stressors of freshwater biodiversity patterns across different ecosystems and scales: A review. Hydrobiologia, 696(1), 1–28. 10.1007/s10750-012-1183-0

[ece310896-bib-0070] Taft, B. , & Koncelik, J. P. (2006). Methods for assessing habitat in flowing waters: Using the qualitative habitat evaluation index (QHEI). Ohio Environmental Protection Agency.

[ece310896-bib-0071] The State Environmental Protection Administration . (2002). Water and wastewater monitoring analysis method. China Environmental Science Press.

[ece310896-bib-0072] Thompson, P. L. , Guzman, L. M. , De Meester, L. , Horvath, Z. , Ptacnik, R. , Vanschoenwinkel, B. , Viana, D. S. , & Chase, J. M. (2020). A process‐based metacommunity framework linking local and regional scale community ecology. Ecology Letters, 23(9), 1314–1329. 10.1111/ele.13568 32672410 PMC7496463

[ece310896-bib-0073] Tonkin, J. D. , Altermatt, F. , Finn, D. S. , Heino, J. , Olden, J. D. , Pauls, S. U. , & Lytle, D. A. (2018). The role of dispersal in river network metacommunities: Patterns, processes, and pathways. Freshwater Biology, 63(1), 141–163. 10.1111/fwb.13037

[ece310896-bib-0074] Van De Meutter, F. , De Meester, L. , & Stoks, R. (2007). Metacommunity structure of pond macro invertebrates: Effects of dispersal mode and generation time. Ecology, 88(7), 1687–1695. 10.1890/06-0333.1 17645015

[ece310896-bib-0075] Varnosfaderany, M. N. , Ebrahimi, E. , Mirghaffary, N. , & Safyanian, A. (2010). Biological assessment of the Zayandeh Rud River, Iran, using benthic macroinvertebrates. Limnologica, 40(3), 226–232. 10.1016/j.limno.2009.10.002

[ece310896-bib-0076] Vilmi, A. , Karjalainen, S. M. , Hellsten, S. , & Heino, J. (2016). Bioassessment in a metacommunity context: Are diatom communities structured solely by species sorting? Ecological Indicators, 62, 86–94. 10.1016/j.ecolind.2015.11.043

[ece310896-bib-0077] Wang, L. , Zhang, R. Q. , Yang, J. , Chen, Q. W. , He, M. N. , & Wang, J. (2021). A method to determine water quality categories based on biotic index of macroinvertebrates in the Yangtze River Delta. Ecological Informatics, 66, 101484. 10.1016/j.ecoinf.2021.101484

[ece310896-bib-0078] Wang, X. , Wang, Q. , Wu, C. , Liang, T. , Zheng, D. , & Wei, X. (2012). A method coupled with remote sensing data to evaluate non‐point source pollution in the Xin'anjiang catchment of China. Science of the Total Environment, 430, 132–143. 10.1016/j.scitotenv.2012.04.052 22634560

[ece310896-bib-0079] Wu, J. , Mao, R. , Li, M. , Xia, J. , Song, J. , Cheng, D. , & Sun, H. (2020). Assessment of aquatic ecological health based on determination of biological community variability of fish and macroinvertebrates in the Weihe River basin, China. Journal of Environmental Management, 267, 110651. 10.1016/j.jenvman.2020.110651 32349958

[ece310896-bib-0080] Wu, N. , Guo, K. , Zou, Y. , He, F. , & Riis, T. (2023). SER: An R package to characterize environmental regimes. Ecology and Evolution, 13(3), e9882. 10.1002/ece3.9882 36919015 PMC10008288

[ece310896-bib-0081] Wu, N. , Liu, G. , Zhang, M. , Wang, Y. , Peng, W. , & Qu, X. (2022). Spatial factors outperform local environmental and geo‐climatic variables in structuring multiple facets of stream Macroinvertebrates' beta‐diversity. Animals, 12(19), 2648. 10.3390/ani12192648 36230389 PMC9558512

[ece310896-bib-0082] Wu, N. , Schmalz, B. , & Fohrer, N. (2012). Development and testing of a phytoplankton index of biotic integrity (P‐IBI) for a German lowland river. Ecological Indicators, 13(1), 158–167. 10.1016/j.ecolind.2011.05.022

[ece310896-bib-0083] Yang, J. , & Huang, X. (2021). The 30 m annual land cover dataset and its dynamics in China from 1990 to 2019. Earth System Science Data, 13(8), 3907–3925. 10.5194/essd-13-3907-2021

[ece310896-bib-0084] Yang, Y. , Wu, Q. , Yang, W. , Wu, F. , Zhang, L. , Xu, Z. , Liu, Y. , Tan, B. , Li, H. , & Zhou, W. (2020). Temperature and soil nutrients drive the spatial distributions of soil macroinvertebrates on the eastern Tibetan plateau. Ecosphere, 11(3), e03075. 10.1002/ecs2.3075

[ece310896-bib-0085] Yorulmaz, B. , & Ertas, A. (2021). Water quality assessment of Selendi stream and comparative performance of the indices based on benthic macroinvertebrates and physicochemical parameters. Biologia, 76(9), 2599–2607. 10.1007/s11756-021-00756-3

[ece310896-bib-0086] You, Q. , Yang, W. , Jian, M. , & Hu, Q. (2021). A comparison of metric scoring and health status classification methods to evaluate benthic macroinvertebrate‐based index of biotic integrity performance in Poyang Lake wetland. Science of the Total Environment, 761, 144112. 10.1016/j.scitotenv.2020.144112 33360123

[ece310896-bib-0087] Young, S.‐S. , Yang, H.‐N. , Huang, D.‐J. , Liu, S.‐M. , Huang, Y.‐H. , Chiang, C.‐T. , & Liu, J. W. (2014). Using benthic macroinvertebrate and fish communities as bioindicators of the Tanshui River basin around the greater Taipei area – Multivariate analysis of spatial variation related to levels of water pollution. International Journal of Environmental Research and Public Health, 11(7), 7116–7143. 10.3390/ijerph110707116 25026081 PMC4113864

[ece310896-bib-0088] Zeybek, M. (2017). Macroinvertebrate‐based biotic indices for evaluating the water quality of Kargi stream (Antalya, Turkey). Turkish Journal of Zoology, 41(3), 476–486. 10.3906/zoo-1602-10

[ece310896-bib-0089] Zhai, X. , Zhang, Y. , Wang, X. , Xia, J. , & Liang, T. (2014). Non‐point source pollution modelling using soil and water assessment tool and its parameter sensitivity analysis in Xin'anjiang catchment, China. Hydrological Processes, 28(4), 1627–1640. 10.1002/hyp.9688

[ece310896-bib-0090] Zhang, J. , Jiang, P. , Chen, K. , He, S. , Wang, B. , & Jin, X. (2020). Development of biological water quality categories for streams using a biotic index of macroinvertebrates in the Yangtze River Delta, China. Ecological Indicators, 117, 106650. 10.1016/j.ecolind.2020.106650

[ece310896-bib-0091] Zhao, C. , Shao, N. , Yang, S. , Ren, H. , Ge, Y. , Zhang, Z. , Zhao, Y. , & Yin, X. (2019). Integrated assessment of ecosystem health using multiple indicator species. Ecological Engineering, 130, 157–168. 10.1016/j.ecoleng.2019.02.016

