# Supplementary material for: Comparison of different macroinvertebrates bioassessment indices in a large near‐natural watershed under the context of metacommunity theory
Source: Ecol Evol. 2024 Feb 5;14(2):e10896. doi: 10.1002/ece3.10896 (PMC10844709; doi:10.1002/ece3.10896)
Supplement: Supplementary file 1 — Appendix S1: [file ECE3-14-e10896-s001.docx]

SUPPORTING INFORMATION

**Comparison of different macroinvertebrates bioassessment indices in a large near-natural watershed under the context of metacommunity theory**

Guohao Liu^1,2^, Xinxin Qi^1,2^, Zongwei Lin^1,2^, Yuanyuan Lv^1,2^, Sangar Khan^1,2^, Xiaodong Qu^3^, Binsong Jin^4^, Ming Wu^5^, Collins Oduro^1,2^, Naicheng Wu^1,2*^

^1^ Department of Geography and Spatial Information Techniques, Ningbo University, Ningbo 315211, China

^2^ Zhejiang Collaborative Innovation Center & Ningbo Universities Collaborative Innovation Center for Land and Marine Spatial Utilization and Governance Research, Ningbo University, Ningbo 315211, China

^3^ State Key Laboratory of Simulation and Regulation of Water Cycle in River Basin, China Institute of Water Resources and Hydropower Research, Beijing 100038, China

^4^ College of Life and Environmental Sciences, Hangzhou Normal University, Hangzhou 311121, China

^5^ Wetland Ecosystem Research Station of Hangzhou Bay, Research Institute of Subtropical Forestry, Chinese Academy of Forestry, Hangzhou 311400, Zhejiang, China

*^*^ Corresponding author: Naicheng Wu (naichengwu88@gmail.com)*


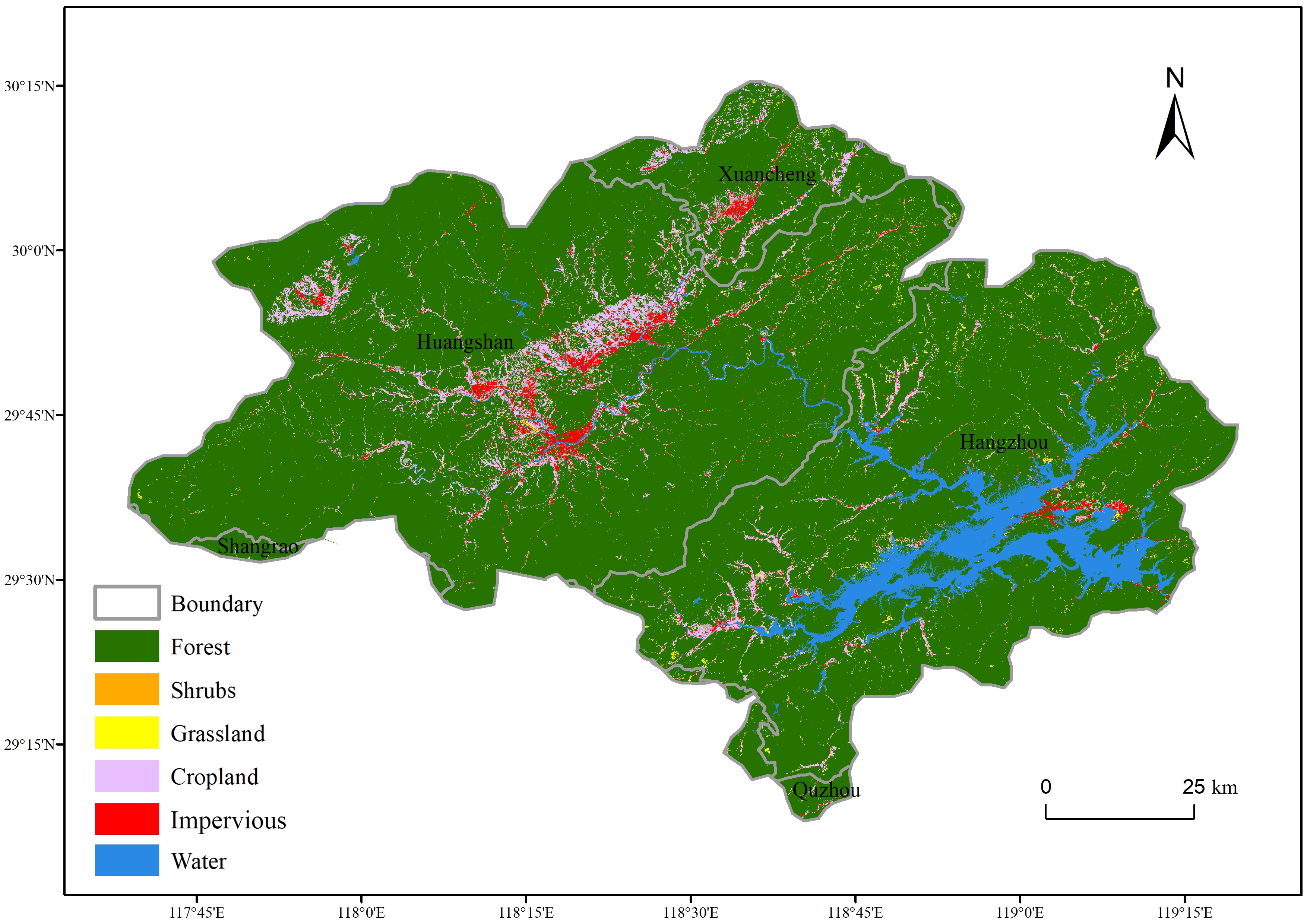


**Figure S1.** Map of land use types in the Thousand Island Lake catchment, China.

**Table S1.** Species of macroinvertebrate identified in this study and their dispersal ability group (DAG) and sensitivity value (SV). DAG1 was mainly comprised of Oligochaeta, Hirudinea, Gastropoda, Bivalvia, and Crustacea; DAG2 was mainly comprised of Ceratopogonidae, Chironomidae; DAG3 was mainly comprised of Ephemeroptera, Plecoptera, Megaloptera, Trichoptera, Diptera; DAG4 was mainly comprised of Odonata, Heteroptera: Corixidae.

| **Species code** | **Phylum** | **Class** | **Order** | **Family** | **Species name** | **DAG** | | **SV** |
| --- | --- | --- | --- | --- | --- | --- | --- | --- |
| SM001 | Annelida | Clitellata | Arhynchobdellida | Erpobdellidae | *Erpobdella* sp. | | 1 | 9 |
| SM002 | Annelida | Clitellata | Arhynchobdellida | Erpobdellidae | *Erpobdellidae* sp. | | 1 | 8 |
| SM003 | Annelida | Clitellata | Haplotaxida | Enchytraeidae | *Marionina* sp. | | 1 | 9 |
| SM004 | Annelida | Hirudinea | Rhynchobdellidae | Glossiphoniidae | *Actinobdella* sp. | | 1 | 8 |
| SM005 | Annelida | Hirudinea | Rhynchobdellidae | Glossiphoniidae | *Helobdella* sp. | | 1 | 8 |
| SM006 | Annelida | Hirudinea | Rhynchobdellidae | Glossiphoniidae | *Glossiphoniidae* sp. | | 1 | 8 |
| SM007 | Annelida | Oligochaeta | Tubificida | Nadidae | *Branchiura sowerbyi* | | 1 | 6 |
| SM008 | Annelida | Oligochaeta | Tubificida | Nadidae | *Limnodrilus hoffmeisteri* | | 1 | 10 |
| SM009 | Annelida | Oligochaeta | Tubificida | Nadidae | *Monopylephorus limosus* | | 1 | 7 |
| SM010 | Annelida | Oligochaeta | Tubificida | [Naididae](https://en.wikipedia.org/wiki/Naididae) | *Tubifex* sp.1 | | 1 | 7 |
| SM011 | Arthropoda | Arachnida | Araneae | Dictynidae | *Argyroneta aquatica* | | 2 | 6 |
| SM012 | Arthropoda | Arachnida | Trombidiformes | Hygrobatidae | *Hygrobatidae* sp. | |  | 6 |
| SM013 | Arthropoda | Arachnida | Trombidiformes | Lebertiidae | *Lebertiidae* sp. | |  | 6 |
| SM014 | Arthropoda | Insecta | Coleptera | Dytiscidae | *Agabus* sp.1 | | 4 | 5 |
| SM015 | Arthropoda | Insecta | Coleptera | Dytiscidae | *Agabus* sp.2 | | 4 | 5 |
| SM016 | Arthropoda | Insecta | Coleptera | Dytiscidae | *Dytiscidae* sp. | | 4 | 5 |
| SM017 | Arthropoda | Insecta | Coleptera | Elmidae | *Ancyronyx variegatus* | | 2 | 5 |
| SM018 | Arthropoda | Insecta | Coleptera | Elmidae | *Dubiraphia* sp.1 | | 2 | 5 |
| SM019 | Arthropoda | Insecta | Coleptera | Elmidae | *Dubiraphia* sp.2 | | 2 | 5 |
| SM020 | Arthropoda | Insecta | Coleptera | Elmidae | *Elmidae* sp.1 | | 2 | 5 |
| SM021 | Arthropoda | Insecta | Coleptera | Elmidae | *Macronychus glabratus* | | 2 | 3 |
| SM022 | Arthropoda | Insecta | Coleptera | Elmidae | *Elmidae* sp.2 | | 2 | 3 |
| SM023 | Arthropoda | Insecta | Coleptera | Elmidae | *Elmidae* sp.3 | | 2 | 4 |
| SM024 | Arthropoda | Insecta | Coleptera | Elmidae | *Optioservus* sp. | | 2 | 4 |
| SM025 | Arthropoda | Insecta | Coleptera | Elmidae | *Stenelmis* sp. | | 2 | 5 |
| SM026 | Arthropoda | Insecta | Coleptera | Gyrinidae | *Dineutus* sp.1 | | 2 | 4 |
| SM027 | Arthropoda | Insecta | Coleptera | Gyrinidae | *Dineutus* sp.2 | | 2 | 4 |
| SM028 | Arthropoda | Insecta | Coleptera | Gyrinidae | *Gyrinus* sp. | | 2 | 4 |
| SM029 | Arthropoda | Insecta | Coleptera | Hydrophilidae | *Hydrophilus* sp. | | 2 | 4 |
| SM030 | Arthropoda | Insecta | Coleptera | Psephenidae | *Psephenus* sp.1 | | 2 | 4 |
| SM031 | Arthropoda | Insecta | Coleptera | Psephenidae | *Psephenus* sp.2 | | 2 | 4 |
| SM032 | Arthropoda | Insecta | Coleptera | Ptilodactylidae | *Ptilodactylidae* sp. | | 2 | 3 |
| SM033 | Arthropoda | Insecta | Coleptera | Scirtidae | *Sacodes* sp. | | 2 | 5 |
| SM034 | Arthropoda | Insecta | Corixidae | Corixidae | *Corixidae* sp. | | 4 | 5 |
| SM035 | Arthropoda | Insecta | Diptera | Athericidae | *Atherix* sp. | |  | 4 |
| SM036 | Arthropoda | Insecta | Diptera | Ceratopogonidae | *Bezzia* sp. | | 2 | 6 |
| SM037 | Arthropoda | Insecta | Diptera | Ceratopogonidae | *Dasyhelea* sp. | | 2 | 6 |
| SM038 | Arthropoda | Insecta | Diptera | Cerattopogonidae | *Probezzia* sp. | | 2 | 6 |
| SM039 | Arthropoda | Insecta | Diptera | Chironomidae | *Chironomus* sp.1 | | 2 | 10 |
| SM040 | Arthropoda | Insecta | Diptera | Chironomidae | *Chironomus* sp.2 | | 2 | 10 |
| SM041 | Arthropoda | Insecta | Diptera | Chironomidae | *Cladopelma* sp. | | 2 | 9 |
| SM042 | Arthropoda | Insecta | Diptera | Chironomidae | *Cladotanytarsus* sp. | | 2 | 5 |
| SM043 | Arthropoda | Insecta | Diptera | Chironomidae | *Clinotarypus* sp. | | 2 | 8 |
| SM044 | Arthropoda | Insecta | Diptera | Chironomidae | *Cricotopus albiforceps* | | 2 | 7 |
| SM045 | Arthropoda | Insecta | Diptera | Chironomidae | *Diamesa* sp. | | 2 | 5 |
| SM046 | Arthropoda | Insecta | Diptera | Chironomidae | *Lipiniella* sp. | | 2 | 6 |
| SM047 | Arthropoda | Insecta | Diptera | Chironomidae | *Orthocladius* sp. | | 2 | 6 |
| SM048 | Arthropoda | Insecta | Diptera | Chironomidae | *Parachaenocladius* sp. | | 2 | 4 |
| SM049 | Arthropoda | Insecta | Diptera | Chironomidae | *Procladius* sp. | | 2 | 9 |
| SM050 | Arthropoda | Insecta | Diptera | Chironomidae | *Thienemannimyia* sp. | | 2 | 6 |
| SM051 | Arthropoda | Insecta | Diptera | [Dolichopodidae](https://en.wikipedia.org/wiki/Dolichopodidae) | *Rhaphium* sp. | | 2 | 4 |
| SM052 | Arthropoda | Insecta | Diptera | Empididae | *Hemerodromia* sp.*.* | | 3 | 6 |
| SM053 | Arthropoda | Insecta | Diptera | Ephydridae | *Hydrellia* sp. | | 2 | 6 |
| SM054 | Arthropoda | Insecta | Diptera | Ephydridae | *Scatella* sp. | | 2 | 6 |
| SM055 | Arthropoda | Insecta | Diptera | Limoniidae | *Antocha* sp. | | 2 | 4.6 |
| SM056 | Arthropoda | Insecta | Diptera | Limoniidae | *Hexatoma* sp.1 | | 2 | 4.7 |
| SM057 | Arthropoda | Insecta | Diptera | Limoniidae | *Hexatoma* sp.2 | | 2 | 4.7 |
| SM058 | Arthropoda | Insecta | Diptera | Limoniidae | *Limoniidae* sp. | | 2 | 4.6 |
| SM059 | Arthropoda | Insecta | Diptera | Muscidae | *Muscidae* sp. | | 2 | 6 |
| SM060 | Arthropoda | Insecta | Diptera | Pediciidae | *Dicranota* sp. | | 2 | 3 |
| SM061 | Arthropoda | Insecta | Diptera | Psychodidae | *Psychoda* sp. | | 2 | 10 |
| SM062 | Arthropoda | Insecta | Diptera | Ptychopteridae | *Ptychoptera* sp. | | 2 | 9 |
| SM063 | Arthropoda | Insecta | Diptera | Simuliidae | *Simuliidae* sp. | | 2 | 6 |
| SM064 | Arthropoda | Insecta | Diptera | Simuliidae | *Simulium* sp. | | 2 | 6 |
| SM065 | Arthropoda | Insecta | Diptera | Simuliidae | *Simulium* sp.1 | | 2 | 6 |
| SM066 | Arthropoda | Insecta | Diptera | Simuliidae | *Simulium* sp.2 | | 2 | 6 |
| SM067 | Arthropoda | Insecta | Diptera | Tabanidae | *Tabanidae* sp. | | 3 | 6 |
| SM068 | Arthropoda | Insecta | Diptera | Tabanidae | *Tabanus reinwardtii* | | 3 | 6 |
| SM069 | Arthropoda | Insecta | Diptera | Tipulidae | *Angarotipula* sp. | | 2 | 3 |
| SM070 | Arthropoda | Insecta | Diptera | Tipulidae | *Tipula* sp.1 | | 2 | 3 |
| SM071 | Arthropoda | Insecta | Diptera | Tipulidae | *Tipula* sp.2 | | 2 | 6 |
| SM072 | Arthropoda | Insecta | Diptera | Tipulidae | *Tipulidae* sp. | | 2 | 6 |
| SM073 | Arthropoda | Insecta | Ephemeroptera | Ameletidae | *Ameletus* sp.1 | | 3 | 0 |
| SM074 | Arthropoda | Insecta | Ephemeroptera | Ameletidae | *Ameletus* sp.2 | | 3 | 0 |
| SM075 | Arthropoda | Insecta | Ephemeroptera | Baetidae | *Baetiella bispinosa* | | 3 | 5 |
| SM076 | Arthropoda | Insecta | Ephemeroptera | Baetidae | *Baetis* sp. | | 3 | 6 |
| SM077 | Arthropoda | Insecta | Ephemeroptera | Baetidae | *Baetidae* sp. | | 3 | 2 |
| SM078 | Arthropoda | Insecta | Ephemeroptera | Baetidae | *Pseudocloeon* sp. | | 3 | 6 |
| SM079 | Arthropoda | Insecta | Ephemeroptera | Caenidae | *Caenis* sp. | | 3 | 6 |
| SM080 | Arthropoda | Insecta | Ephemeroptera | Ephemerellidae | *Ephemerellidae* sp.1 | | 3 | 1 |
| SM081 | Arthropoda | Insecta | Ephemeroptera | Ephemerellidae | *Drunella* sp. | | 3 | 2 |
| SM082 | Arthropoda | Insecta | Ephemeroptera | Ephemerellidae | *Ephemerella* sp.1 | | 3 | 2 |
| SM083 | Arthropoda | Insecta | Ephemeroptera | Ephemerellidae | *Ephemerella* sp.2 | | 3 | 2 |
| SM084 | Arthropoda | Insecta | Ephemeroptera | Ephemerellidae | *Ephemerellidae* sp.2 | | 3 | 2 |
| SM085 | Arthropoda | Insecta | Ephemeroptera | Ephemerellidae | *Eurylophella* sp. | | 3 | 2 |
| SM086 | Arthropoda | Insecta | Ephemeroptera | Ephemerellidae | *Serratella* sp. | | 3 | 1 |
| SM087 | Arthropoda | Insecta | Ephemeroptera | Ephemeridae | *Ephemera* sp. | | 3 | 2 |
| SM088 | Arthropoda | Insecta | Ephemeroptera | Ephemeridae | *Hexagenia* sp. | | 3 | 6 |
| SM089 | Arthropoda | Insecta | Ephemeroptera | Heptageniidae | *Cinygmina* sp. | | 3 | 2 |
| SM090 | Arthropoda | Insecta | Ephemeroptera | Heptageniidae | *Epeorus melli* | | 3 | 0 |
| SM091 | Arthropoda | Insecta | Ephemeroptera | Heptageniidae | *Epeorus* sp. | | 3 | 0 |
| SM092 | Arthropoda | Insecta | Ephemeroptera | Heptageniidae | *Heptagenia* sp. | | 3 | 4 |
| SM093 | Arthropoda | Insecta | Ephemeroptera | Heptageniidae | *Heptageniidae* sp.1 | | 3 | 4 |
| SM094 | Arthropoda | Insecta | Ephemeroptera | Heptageniidae | *Heptageniidae* sp.2 | | 3 | 4 |
| SM095 | Arthropoda | Insecta | Ephemeroptera | Heptageniidae | *Rhithrogena* sp. | | 3 | 0 |
| SM096 | Arthropoda | Insecta | Ephemeroptera | Heptageniidae | *Heptageniidae* sp.3 | | 3 | 4 |
| SM097 | Arthropoda | Insecta | Ephemeroptera | Heptageniidae | *Thalerosphyrus vietnamensis* | | 3 | 4 |
| SM098 | Arthropoda | Insecta | Ephemeroptera | Isonychiidae | *Isonychia* sp. | | 3 | 2 |
| SM099 | Arthropoda | Insecta | Ephemeroptera | Leptohyphidae | *Tricorythodes* sp. | | 3 | 4 |
| SM100 | Arthropoda | Insecta | Ephemeroptera | Leptophlebiidae | *Leptophlebiidae* sp.1 | | 3 | 4 |
| SM101 | Arthropoda | Insecta | Ephemeroptera | Leptophlebiidae | *Leptophlebiidae* sp.2 | | 3 | 4 |
| SM102 | Arthropoda | Insecta | Ephemeroptera | Leptophlebiidae | *Leptophlebia* sp. | | 3 | 4 |
| SM103 | Arthropoda | Insecta | Ephemeroptera | Leptophlebiidae | *Leptophlebiidae* sp.3 | | 3 | 2 |
| SM104 | Arthropoda | Insecta | Ephemeroptera | Leptophlebiidae | *Leptophlebiidae* sp.4 | | 3 | 2 |
| SM105 | Arthropoda | Insecta | Ephemeroptera | Metretopodidae | *Siphloplecton* sp. | | 3 | 2 |
| SM106 | Arthropoda | Insecta | Ephemeroptera | Polymitarcyidae | *Ephoron* sp. | | 3 | 2 |
| SM107 | Arthropoda | Insecta | Ephemeroptera | Potamanthidae | *Anthopotamus* sp. | | 3 | 4 |
| SM108 | Arthropoda | Insecta | Ephemeroptera | Siphlonuridae | *Siphlonuridae* sp. | | 3 | 4 |
| SM109 | Arthropoda | Insecta | Hemiptera | Belostomatidae | *Belostoma* sp. | | 3 | 8 |
| SM110 | Arthropoda | Insecta | Hemiptera | Gerridae | *Gerris* sp. | | 1 | 5 |
| SM111 | Arthropoda | Insecta | Hemiptera | Gerridae | *Gerridae* sp. | | 2 | 5 |
| SM112 | Arthropoda | Insecta | Hemiptera | Naucoridae | *Naucoridae* | | 2 | 7 |
| SM113 | Arthropoda | Insecta | Hemiptera | Nepidae | *Nepa cinerea* | | 3 | 7.5 |
| SM114 | Arthropoda | Insecta | Lepidoptera | Crambidae | *Parapoynx* sp. | | 3 | 5 |
| SM115 | Arthropoda | Insecta | Megaloptera | Corydalidae | *Corydalidae* sp. | | 3 | 4 |
| SM116 | Arthropoda | Insecta | Megaloptera | Sialidae | *Sialis* sp. | | 3 | 4 |
| SM117 | Arthropoda | Insecta | Odonata | Aeshnidae | *Boyeria* sp. | | 4 | 2 |
| SM118 | Arthropoda | Insecta | Odonata | Calopterygidae | *Calopteryx* sp. | | 4 | 6 |
| SM119 | Arthropoda | Insecta | Odonata | Coenagrionidae | *Argia* sp. | | 4 | 6 |
| SM120 | Arthropoda | Insecta | Odonata | Coenagrionidae | *Enallagma* sp. | | 4 | 8 |
| SM121 | Arthropoda | Insecta | Odonata | Coenagrionidae | *Ischnura* sp. | | 4 | 9 |
| SM122 | Arthropoda | Insecta | Odonata | Cordulegastridae | *Cordulegaster* sp.1 | | 4 | 3 |
| SM123 | Arthropoda | Insecta | Odonata | Cordulegastridae | *Cordulegaster* sp.2 | | 4 | 3 |
| SM124 | Arthropoda | Insecta | Odonata | Corduliidae | *Epitheca* sp.1 | | 4 | 5 |
| SM125 | Arthropoda | Insecta | Odonata | Corduliidae | *Epitheca* sp.2 | | 4 | 5 |
| SM126 | Arthropoda | Insecta | Odonata | Gomphidae | *Lanthus* sp.1 | | 4 | 5 |
| SM127 | Arthropoda | Insecta | Odonata | Gomphidae | *Lanthus* sp.2 | | 4 | 5 |
| SM128 | Arthropoda | Insecta | Odonata | Gomphidae | *Ophiogomphus* sp. | | 4 | 1 |
| SM129 | Arthropoda | Insecta | Odonata | Gomphidae | *Ophiogomphus* sp.2 | | 4 | 1 |
| SM130 | Arthropoda | Insecta | Plecoptera | Capniidae | *Capniidae* sp. | | 3 | 3 |
| SM131 | Arthropoda | Insecta | Plecoptera | Chloroperlidae | *Alloperla* sp.1 | | 3 | 0 |
| SM132 | Arthropoda | Insecta | Plecoptera | Chloroperlidae | *Alloperla* sp.2 | | 3 | 0 |
| SM133 | Arthropoda | Insecta | Plecoptera | Leuctridae | *Leuctra* sp. | | 3 | 0 |
| SM134 | Arthropoda | Insecta | Plecoptera | Perlidae | *Acroneuria* sp. | | 3 | 0 |
| SM135 | Arthropoda | Insecta | Plecoptera | Perlidae | *Agnetina* sp. | | 3 | 2 |
| SM136 | Arthropoda | Insecta | Plecoptera | Perlidae | *Neoperla* sp. | | 3 | 3 |
| SM137 | Arthropoda | Insecta | Plecoptera | Perlidae | *Peragnetina* sp. | | 3 | 2 |
| SM138 | Arthropoda | Insecta | Plecoptera | Perlodidae | *Perlodidae* sp. | | 3 | 2 |
| SM139 | Arthropoda | Insecta | Plecoptera | Perlodidae | *Stavsolus* sp. | | 3 | 2 |
| SM140 | Arthropoda | Insecta | Plecoptera | Styloperlidae | *Styloperla* sp. | | 3 | 1 |
| SM141 | Arthropoda | Insecta | [Trichoptera](https://en.wikipedia.org/wiki/Caddisfly) | Apataniidae | *Apatania* sp. | | 3 | 3 |
| SM142 | Arthropoda | Insecta | Trichoptera | Glossosomatidae | *Glossosoma* sp. | | 3 | 0 |
| SM143 | Arthropoda | Insecta | [Trichoptera](https://en.wikipedia.org/wiki/Caddisfly) | Helicopsychidae | *Helicopsyche* sp. | | 3 | 3 |
| SM144 | Arthropoda | Insecta | Trichoptera | Hydropsychidae | *Arctopsyche* sp. | | 3 | 1 |
| SM145 | Arthropoda | Insecta | Trichoptera | Hydropsychidae | *Cheumatopsyche* sp.1 | | 3 | 5 |
| SM146 | Arthropoda | Insecta | Trichoptera | Hydropsychidae | *Cheumatopsyche* sp.2 | | 3 | 5 |
| SM147 | Arthropoda | Insecta | Trichoptera | Hydropsychidae | *Diplectrona* sp. | | 3 | 5 |
| SM148 | Arthropoda | Insecta | Trichoptera | Hydropsychidae | *Hydropsyche* sp. | | 3 | 4 |
| SM149 | Arthropoda | Insecta | Trichoptera | Hydropsychidae | *Macrostemum* sp. | | 3 | 3 |
| SM150 | Arthropoda | Insecta | Trichoptera | Lepidostomatidae | *Lepidostoma* sp. | | 3 | 1 |
| SM151 | Arthropoda | Insecta | Trichoptera |  | *Trichoptera* sp.1 | |  |  |
| SM152 | Arthropoda | Insecta | Trichoptera | Limnephilidae | *Limnephilidae* sp.1 | | 3 | 4 |
| SM153 | Arthropoda | Insecta | Trichoptera | Limnephilidae | *Limnephilidae* sp.2 | | 3 | 4 |
| SM154 | Arthropoda | Insecta | Trichoptera | Philopotamidae | *Chimarra* sp.1 | | 3 | 4 |
| SM155 | Arthropoda | Insecta | Trichoptera | Philopotamidae | *Chimarra* sp.2 | | 3 | 4 |
| SM156 | Arthropoda | Insecta | Trichoptera | Phryganeidae | *Phryganeida* sp. | | 3 | 4 |
| SM157 | Arthropoda | Insecta | Trichoptera | Polycentropodidae | *Nyctiophylax* sp. | | 3 | 5 |
| SM158 | Arthropoda | Insecta | Trichoptera | Polycentropodidae | *Polycentropus* sp. | | 3 | 6 |
| SM159 | Arthropoda | Insecta | Trichoptera | Polycentropodinae | *Neureclipisis mandjurica* | | 3 | 7 |
| SM160 | Arthropoda | Insecta | Trichoptera | Psychomyiidae | *Psychomyia* sp. | | 3 | 2 |
| SM161 | Arthropoda | Insecta | Trichoptera | Rhyacophilidae | *Rhyacophila* sp. | | 3 | 0 |
| SM162 | Arthropoda | Insecta | Trichoptera |  | *Trichoptera* sp.2 | |  |  |
| SM163 | Arthropoda | Insecta | Trichoptera | Uenoidae | *Neophylax rickeri* | | 3 | 3 |
| SM164 | Arthropoda | Malacostraca | Decapoda | Palaemonidae | *Exopalaemon modestus* | | 1 | 8 |
| SM165 | Arthropoda | Malacostraca | Decapoda | Palaemonidae | *Exopalaemon* sp.1 | | 1 | 8 |
| SM166 | Arthropoda | Malacostraca | Decapoda | Palaemonidae | *Exopalaemon* sp.2 | | 1 | 8 |
| SM167 | Arthropoda | Malacostraca | Decapoda | Sinopotamidae | *Sinopotamon chekiangense* | | 1 | 8 |
| SM168 | Arthropoda | Malacostraca | Decapoda | Sinopotamidae | *Sinopotamon* sp. | | 1 | 8 |
| SM169 | Mollusca | Bivalvia | Veneroida | Corbiculidae | *Corbicula fluminea* | | 1 | 6 |
| SM170 | Mollusca | Bivalvia | Veneroida | Corbiculidae | *Corbicula largillierti* | | 1 | 6 |
| SM171 | Mollusca | Bivalvia | Veneroida | Corbiculidae | *Corbicula nitens* | | 1 | 6 |
| SM172 | Mollusca | Bivalvia | Veneroida | Corbiculidae | *Sphaerium japonicum* | | 1 | 6 |
| SM173 | Mollusca | Gastropoda | Heterostropha | Valvatidae | *Valvata lewisi* | | 1 | 8 |
| SM174 | Mollusca | Gastropoda | Littorinimorpha | Hydrobiidae | *Lithoglyphopsis ovatus* | | 1 | 7 |
| SM175 | Mollusca | Gastropoda | Mesogastropoda | [Bithyniidae](https://en.wikipedia.org/wiki/Bithyniidae) | *Bithynia leachi* | | 1 | 8 |
| SM176 | Mollusca | Gastropoda | Mesogastropoda | Lymnaeidae | *Radix amygdalus* | | 1 | 7 |
| SM177 | Mollusca | Gastropoda | Mesogastropoda | Lymnaeidae | *Radix auricularia* | | 1 | 7 |
| SM178 | Mollusca | Gastropoda | Mesogastropoda | Lymnaeidae | *Radix ovata* | | 1 | 7 |
| SM179 | Mollusca | Gastropoda | Mesogastropoda | Lymnaeidae | *Radix plicatula* | | 1 | 7 |
| SM180 | Mollusca | Gastropoda | Mesogastropoda | Lymnaeidae | *Radix* sp. | | 1 | 7 |
| SM181 | Mollusca | Gastropoda | Mesogastropoda | Planorbidae | *Gyraulus convexiusculus* | | 1 | 6 |
| SM182 | Mollusca | Gastropoda | Mesogastropoda | Planorbidae | *Hippeutis cantori* | | 1 | 6 |
| SM183 | Mollusca | Gastropoda | Mesogastropoda | Planorbidae | *Hippeutis umbilicalis* | | 1 | 6 |
| SM184 | Mollusca | Gastropoda | Mesogastropoda | Semisulcospiridae | *Hua* sp.1 | | 1 | 7 |
| SM185 | Mollusca | Gastropoda | Mesogastropoda | Semisulcospiridae | *Hua* sp.2 | | 1 | 7 |
| SM186 | Mollusca | Gastropoda | Mesogastropoda | Semisulcospiridae | *Koreoleptoxis nodifila* | | 1 | 7 |
| SM187 | Mollusca | Gastropoda | Mesogastropoda | Semisulcospiridae | *Koreoleptoxis peregrinorum* | | 1 | 7 |
| SM188 | Mollusca | Gastropoda | Mesogastropoda | Semisulcospiridae | *Koreoleptoxis* sp.1 | | 1 | 7 |
| SM189 | Mollusca | Gastropoda | Mesogastropoda | Semisulcospiridae | *Koreoleptoxis* sp.2 | | 1 | 7 |
| SM190 | Mollusca | Gastropoda | Mesogastropoda | Semisulcospiridae | *Semisulcospira cancellata* | | 1 | 7 |
| SM191 | Mollusca | Gastropoda | Mesogastropoda | Semisulcospiridae | *Semisulcospira jacquetiana* | | 1 | 7 |
| SM192 | Mollusca | Gastropoda | Mesogastropoda | Semisulcospiridae | *Semisulcospira libertina* | | 1 | 7 |
| SM193 | Mollusca | Gastropoda | Mesogastropoda | Semisulcospiridae | *Semisulcospira* sp. | | 1 | 7 |
| SM194 | Mollusca | Gastropoda | Mesogastropoda | Viviparidae | *Bellamya angularis* | | 1 | 7 |
| SM195 | Mollusca | Gastropoda | Mesogastropoda | Viviparidae | *Bellamya lapillorum* | | 1 | 7 |
| SM196 | Mollusca | Gastropoda | Mesogastropoda | Viviparidae | *Cipangopaludina cathayensis* | | 1 | 6 |
| SM197 | Mollusca | Gastropoda | Mesogastropoda | Viviparidae | *Viviparus quadratus* | | 1 | 7 |
| SM198 | Mollusca | Gastropoda | Mesogastropoda | Viviparidae | *Viviparus viviparus* | | 1 | 7 |
| SM199 | Platyhelminthes | Turbellaria | Tricladida | Dugesiidae | *Dugesia* sp. | | 1 | 9 |

**Table S2.** Spearman rank correlation coefficients among environmental factors. Significance was indicated as ** p < 0.01, * p < 0.05.

|  | Width | Depth | Velocity | WT | pH | Cond | CODMn | NH3-N | NO3-N | TN | TP | QHEI | Elevation | Slope | Aspect | Forest | Cropland | Shrubs | Grassland | Water | Impervious |
| --- | --- | --- | --- | --- | --- | --- | --- | --- | --- | --- | --- | --- | --- | --- | --- | --- | --- | --- | --- | --- | --- |
| Width | 1.000 |  |  |  |  |  |  |  |  |  |  |  |  |  |  |  |  |  |  |  |  |
| Depth | 0.207^*^ | 1.000 |  |  |  |  |  |  |  |  |  |  |  |  |  |  |  |  |  |  |  |
| Velocity | -0.147 | -0.083 | 1.000 |  |  |  |  |  |  |  |  |  |  |  |  |  |  |  |  |  |  |
| WT | 0.185^*^ | 0.082 | -0.126 | 1.000 |  |  |  |  |  |  |  |  |  |  |  |  |  |  |  |  |  |
| pH | -0.124 | 0.022 | -0.051 | 0.729^**^ | 1.000 |  |  |  |  |  |  |  |  |  |  |  |  |  |  |  |  |
| Cond | 0.300^**^ | 0.013 | -0.147 | -.237^**^ | -.324^**^ | 1.000 |  |  |  |  |  |  |  |  |  |  |  |  |  |  |  |
| CODMn | 0.196^*^ | 0.182^*^ | -0.016 | -.264^**^ | -.350^**^ | 0.294^**^ | 1.000 |  |  |  |  |  |  |  |  |  |  |  |  |  |  |
| NH3-N | 0.160 | 0.003 | 0.002 | -.175^*^ | -0.035 | 0.125 | 0.308^**^ | 1.000 |  |  |  |  |  |  |  |  |  |  |  |  |  |
| NO3-N | -0.069 | -0.090 | 0.128 | -0.076 | 0.098 | 0.255^**^ | 0.079 | 0.093 | 1.000 |  |  |  |  |  |  |  |  |  |  |  |  |
| TN | 0.007 | -0.035 | 0.104 | -0.033 | 0.074 | 0.342^**^ | 0.151 | 0.103 | 0.893^**^ | 1.000 |  |  |  |  |  |  |  |  |  |  |  |
| TP | 0.347^**^ | 0.025 | -0.068 | -0.164 | -0.285^**^ | 0.452^**^ | 0.260^**^ | 0.204^*^ | 0.205^*^ | 0.311^**^ | 1.000 |  |  |  |  |  |  |  |  |  |  |
| QHEI | 0.026 | 0.179^*^ | 0.217^**^ | -0.147 | -0.034 | -0.229^**^ | 0.091 | -0.021 | 0.056 | -0.006 | -0.217^**^ | 1.000 |  |  |  |  |  |  |  |  |  |
| Elevation | -0.488^**^ | -0.189^*^ | 0.143 | -0.547^**^ | -0.321^**^ | -0.277^**^ | -0.001 | -0.127 | -0.231^**^ | -0.290^**^ | -0.234^**^ | 0.272^**^ | 1.000 |  |  |  |  |  |  |  |  |
| Slope | -0.338^**^ | -0.117 | 0.224^**^ | -0.133 | 0.068 | -0.344^**^ | -0.131 | -0.177^*^ | 0.041 | -0.073 | -0.284^**^ | 0.278^**^ | 0.436^**^ | 1.000 |  |  |  |  |  |  |  |
| Aspect | -0.117 | 0.031 | -0.003 | -0.133 | -0.049 | -0.069 | 0.027 | -0.088 | -0.005 | 0.008 | 0.040 | 0.045 | 0.112 | 0.114 | 1.000 |  |  |  |  |  |  |
| Forest | -0.361^**^ | -0.115 | 0.175^*^ | -0.349^**^ | -0.130 | -0.539^**^ | -0.233^**^ | -0.266^**^ | -0.305^**^ | -0.372^**^ | -0.442^**^ | 0.318^**^ | 0.690^**^ | 0.496^**^ | 0.096 | 1.000 |  |  |  |  |  |
| Cropland | 0.344^**^ | 0.107 | -0.180^*^ | 0.346^**^ | 0.119 | 0.529^**^ | 0.234^**^ | 0.263^**^ | 0.293^**^ | 0.358^**^ | 0.442^**^ | -0.331^**^ | -0.689^**^ | -0.496^**^ | -0.108 | -0.995^**^ | 1.000 |  |  |  |  |
| Shrubs | 0.301^**^ | 0.175^*^ | 0.058 | 0.169^*^ | 0.143 | -0.009 | -0.058 | -0.122 | 0.220^**^ | 0.177^*^ | -0.003 | 0.359^**^ | -0.176^*^ | -0.033 | 0.121 | -0.150 | 0.113 | 1.000 |  |  |  |
| Grassland | 0.296^**^ | 0.065 | 0.052 | 0.248^**^ | 0.228^**^ | 0.027 | -0.051 | -0.044 | 0.246^**^ | 0.209^*^ | 0.046 | 0.193^*^ | -0.255^**^ | 0.045 | -0.040 | -.173^*^ | 0.121 | 0.604^**^ | 1.000 |  |  |
| Water | 0.211^*^ | 0.178^*^ | -0.143 | 0.165^*^ | 0.010 | 0.313^**^ | 0.306^**^ | 0.303^**^ | 0.071 | 0.173^*^ | 0.322^**^ | -0.177^*^ | -0.401^**^ | -0.448^**^ | -0.006 | -0.591^**^ | 0.574^**^ | 0.056 | 0.124 | 1.000 |  |
| Impervious | 0.465^**^ | 0.157 | -0.204^*^ | 0.428^**^ | 0.222^**^ | 0.479^**^ | 0.116 | 0.196^*^ | 0.254^**^ | 0.328^**^ | 0.429^**^ | -0.233^**^ | -0.717^**^ | -0.475^**^ | -0.073 | -0.853^**^ | 0.824^**^ | 0.211^*^ | 0.362^**^ | 0.584^**^ | 1.000 |

**Table S3.** Spearman rank correlation coefficients among macroinvertebrates bioassessment indices. Significance was indicated as ** p < 0.01.

| **Indices** | **H'** | **BMWP** | **HBI** | **ASPT** | **EPT-Fa** |
| --- | --- | --- | --- | --- | --- |
| H' | 1 |  |  |  |  |
| BMWP | 0.566** | 1 |  |  |  |
| HBI | -0.266** | -0.320** | 1 |  |  |
| ASPT | 0.248** | 0.632** | -0.408** | 1 |  |
| EPT-Fa | 0.572** | 0.905** | -0.370** | 0.577** | 1 |

H': Shannon Weiner diversity index; BMWP: Biological Monitoring Working Party; ASPT: average score per taxon; BI: biotic index; EPT: EPT taxa index.
